# Current Challenges in Microcapsule Designs and Microencapsulation Processes: A Review

**DOI:** 10.1021/acsami.4c02462

**Published:** 2024-07-23

**Authors:** Benjamin T. Lobel, Daniele Baiocco, Mohammed Al-Sharabi, Alexander F. Routh, Zhibing Zhang, Olivier J. Cayre

**Affiliations:** † School of Chemical and Process Engineering, University of Leeds, Woodhouse LS2 9JT, United Kingdom; ‡ School of Chemical Engineering, University of Birmingham, Birmingham B15 2TT, United Kingdom; § Department of Chemical Engineering and Biotechnology, University of Cambridge, Cambridge CB3 0AS, United Kingdom

**Keywords:** microencapsulation, active ingredient delivery, sustainable microcapsule design, microcapsule fabrication

## Abstract

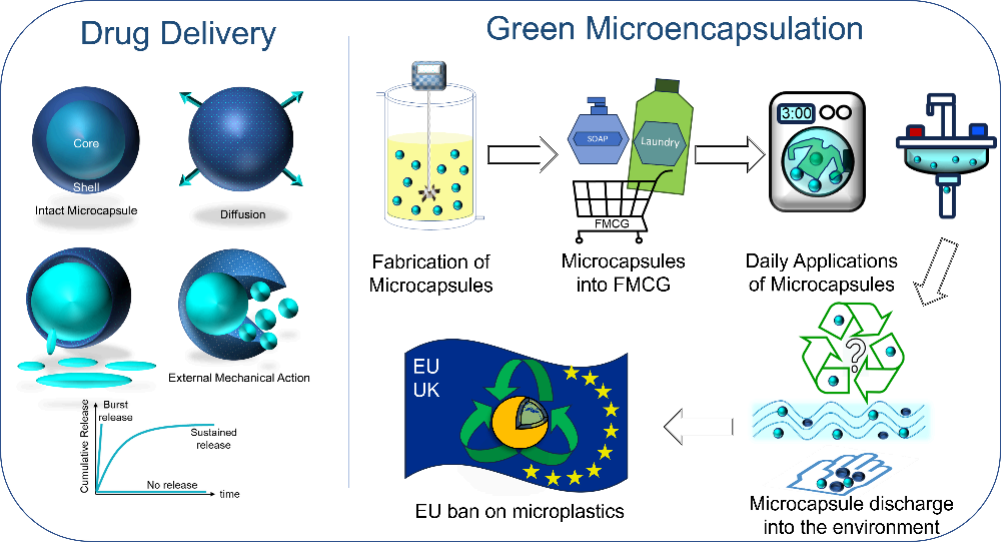

Microencapsulation is an advanced methodology for the protection, preservation, and/or delivery of active materials in a wide range of industrial sectors, such as pharmaceuticals, cosmetics, fragrances, paints, coatings, detergents, food products, and agrochemicals. Polymeric materials have been extensively used as microcapsule shells to provide appropriate barrier properties to achieve controlled release of the encapsulated active ingredient. However, significant limitations are associated with such capsules, including undesired leaching and the nonbiodegradable nature of the typically used polymers. In addition, the energy cost of manufacturing microcapsules is an important factor to be considered when designing microcapsule systems and the corresponding production processes. Recent factors linked to UN sustainability goals are modifying how such microencapsulation systems should be designed in pursuit of “ideal” microcapsules that are efficient, safe, cost-effective and environmentally friendly. This review provides an overview of advances in microencapsulation, with emphasis on sustainable microcapsule designs. The key evaluation techniques to assess the biodegradability of microcapsules, in compliance with recently evolving European Union requirements, are also described. Moreover, the most common methodologies for the fabrication of microcapsules are presented within the framework of their energy demand. Recent promising microcapsule designs are also highlighted for their suitability toward meeting current design requirements and stringent regulations, tackling the ongoing challenges, limitations, and opportunities.

## 1 Introduction

Microencapsulation is a rapidly expanding technology in many consumer goods to protect and deliver active ingredients at end-use applications.^1,2^ Specifically, active ingredients i.e. solid materials, liquid droplets^3^ or gaseous molecules^4^ are entrapped within an inert shell consisting of synthetic,^5^ and/or bioinspired,^6,7^ materials, which are able to segregate chemically and thermally unstable active ingredients from adverse environmental conditions (*e.g*. light, oxidation, and pH changes).^6,8^ The earliest microencapsulation technologies arose during the first half of the twentieth century with the fabrication of gelatin-gum Arabic microcapsules via complex coacervation for carbonless copying paper.^9^

Commercial microcapsules have mostly been engineered with a core–shell configuration where an inert encapsulant (i.e., the shell) acts dually to protect the active ingredients and administer their release, either gradually or instantaneously.^10,11^ Accordingly, the development of a specific micro delivery system is a complex process that entails the actualisation of the targeted active ingredient release mechanism conditionally upon the desired end-use applications.^12^

**Figure 1:**
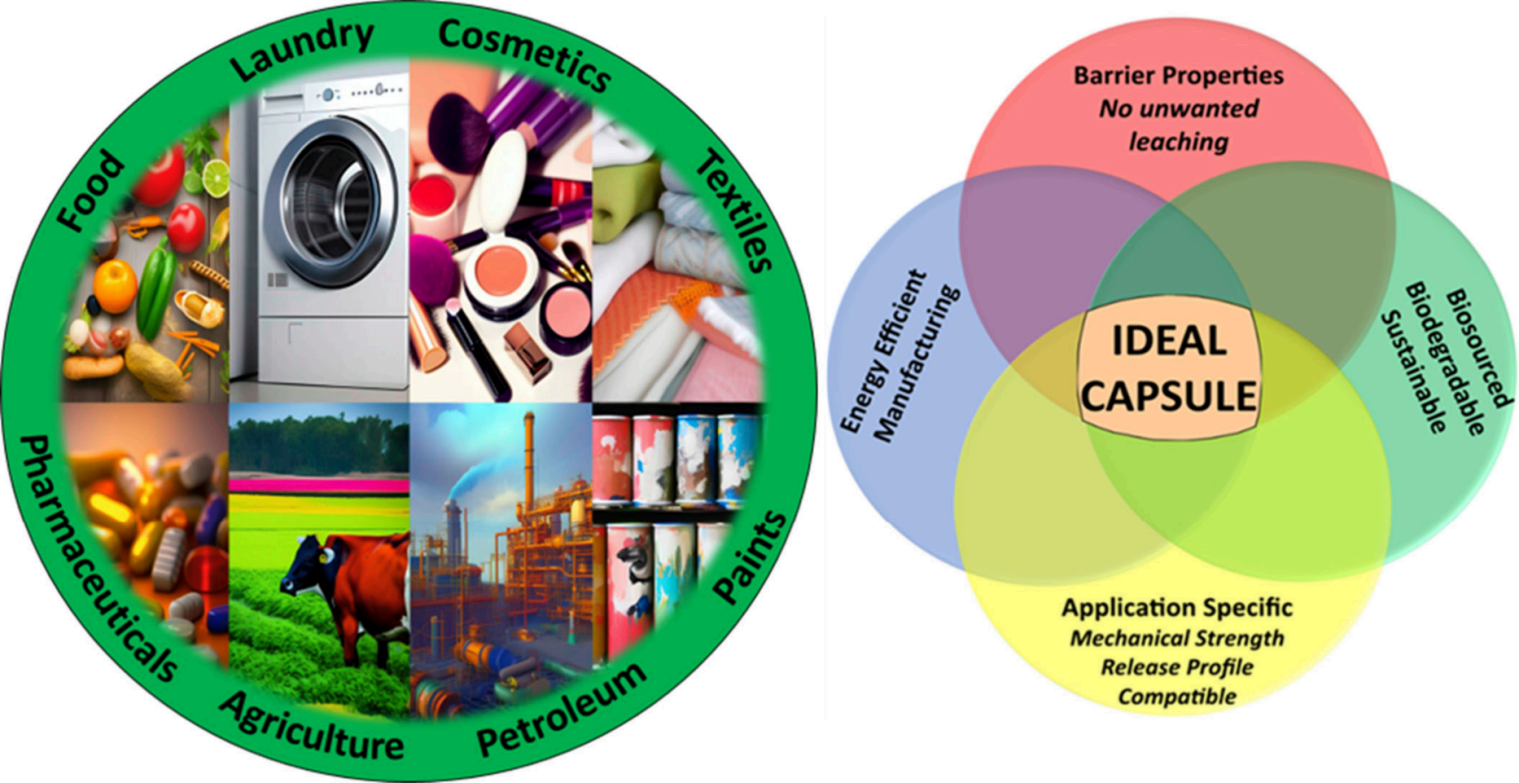
Current footing of the microencapsulation sector. Left: Examples of current industrial applications of microencapsulation technologies. Right: Current main challenges for formulators to consider in developing an ideal microcapsule for these various applications.

The protection and retention of active ingredients within the core of microcapsules and the subsequent control of their release has provided a potential design choice to increase efficiency in formulated products. Indeed, a wide range of applications are now routinely using encapsulation methods for reducing active ingredient dosages or for providing enhanced product performance. This applies to many industries including, but not limited to, agrochemicals, paints and coatings, cosmetics, home and personal care, foods, nutraceuticals, and pharmaceuticals (Figure 1).^13−23^

The implementation of microcapsules in these products and their associated benefits for product performance and dosage reduction can still be drastically improved. When referring to an “ideal” microcapsule this is of course application specific. In many cases a prolonged release may be required, for example in fragrances and pharmaceuticals. In others a burst release may be advantageous–flavourings, detergents, self-healing cement, while in the case of, for example, phase change materials no release at all may be considered ideal. However, an ideal microcapsule must demonstrate a response to external stimuli. More specifically, release, if desired, should ideally not occur until the internal contents of the capsule are required at the intended target–whether this release is then burst or sustained is secondary. Capsules should ideally be biosourced and/or biodegradable and be commercially and energetically efficient to manufacture. While many current capsules reported in the academic landscape are able to increase their rate of release based on temperature, pH or mechanical agitation,^24−29^ very few capsules demonstrate zero release until being triggered.^30^ This is imperative for long-term storage or “shelf-life” of a microcapsule-based product (a key concern in the potential pathway for translation to commercialization), but is difficult to achieve using the characteristics of polymeric shells that many capsules currently available in industrial and academic literature rely on.^5,31−35^ A closely related aspect is the fate of spent microcapsules after their use. Increasingly, microcapsule components of formulated products are being scrutinized to adapt to the current product formulation requirements driven by modern industrial challenges and the United Nations Sustainable Development Goals, such as minimizing product waste and plastic pollution in consideration of overall product lifecycle. Importantly, the recent impending EU regulation around the use of microplastics (MPs) in formulated products has been driving the need for designing biodegradable microcapsules.^36,37^ These environmental issues are compounded by the need to reduce energy consumption and increase manufacturing efficiency in the context of a global energy crisis.^38,39^

Despite significant progress in both the academic and patent literature, as far as the authors are aware, the ideal microcapsule design (controlled retention, biodegradable, application specific, and energy efficient manufacturing/processing, Figure 1) has not yet been demonstrated.^5,12,14,19,31,32,40−49^ As a result, there is renewed focus in the pursuit of the ideal microcapsule design, particularly as a result of the new regulations driving changes in the types of polymers used as part of capsule designs.^13,50−52^ Indeed, an ideal design would allow for the preparation of size-controlled microcapsules where a) leaching is entirely prevented in the formulated product until active ingredient release is activated and delivered to the target site efficiently when the product is in use, b) all of the capsule material is easily biodegraded after use and c) production at industrial scale is energetically efficient and commercially viable.

This article reviews recent advancements in addressing these design requirements and examines the methods used to evaluate their effectiveness. In particular, examples of biocompatible or biodegradable microcapsules in the literature are scrutinized with respect to the current industrial or incoming legislative requirements. Indeed, claims of biodegradable systems are sometimes made on the basis of the biodegradation properties of the polymers used in the microcapsule designs, which does not provide sufficient evidence within the new/upcoming regulation framework. Thus, here we evaluate both the capsules and how their biodegradability can be tested. In addition, the article addresses the industrial challenge toward reducing energy consumption in producing microcapsules, particularly their emulsion precursors. We aim to highlight design examples that attempt to integrate solutions for more than one of these challenges. In a final section, we bring all these advances together to understand what designs may be most promising in the quest to produce microcapsules within the context of current formulated product requirements. Within the evolving legislation regarding these systems, we also propose potential optimization pathways toward developing next generation microcapsule designs. Importantly, this review critically contextualises the current challenges in microencapsulation, drawing on well-established diffusion theories and release methodologies, placing special emphasis on biodegradability, impending legislation, regulation, the perceived horizon and future directions of research in this area. For additional information regarding these fundamental concepts the reader is directed to the following review papers.^53−56^

## 2 Barrier and Release Properties

### 2.1 Polymeric Shells

The permeable nature of polymeric films, and the corresponding microcapsule shells formed from these materials, means that complete retention of small molecules is extremely difficult.^31,57,58^ However, this does not mean that polymer shells are ineffective at active ingredient retention for industrial applications, simply that, instead, there is a threshold of acceptable active ingredient loss in the product. Currently, polymeric microcapsules are used in a variety of industries such as cosmetics, pharmaceuticals, nutraceuticals, food and homecare products.^59^ These microcapsules are formulated in a variety of structural configurations (core–shell, multinuclear, matrix embedded).^60^ Synthetic polymers, such as melamine-based resins,^61^ polyurethane-urea,^62^ poly(methyl methacrylate) (PMMA)^35,63^ and aliphatic polyesters^64,65^ have been extensively used to form microcapsule walls owing to their excellent thermochemical stability, nontoxicity, and excellent elasto-mechanical properties.^66^ These capsules exhibit some loss of their core materials during storage that are deemed acceptable and are accounted for in product development–i.e. their intended effect is still achievable. While this permeability is inherent to such polymeric materials (Table 1), it can be mitigated in a variety of ways. Some of these include the use of cross-linking agents that form molecular bonds between polymeric chains to increase their structural integrity, a chemical response that increases/decreases pore size or adapting the chemistry of the capsule wall to prevent active ingredient permeation into the continuous phase–i.e. preferential wetting of the capsule wall. Alternatively, increasing the polymer shell thickness, either by simply increasing mass or by utilizing a layer-by-layer (LbL) approach will also diminish the release of an active into the surrounding environment (to a limited degree).

**Table 1:** Summary of Polymeric Capsules Presented in Section 2.1

core	shell	size (μm)	proposed application(s)	year	reference
Toluene and coumarin	Polyampholyte	200–350	Fragrance, agriculture	2017	(27)
Pepsin	poly(propylene oxide) and polymethacrylic acid	169	Not Reported	2023	(72)
Rhodamine 6G	Polystyrene-poly(2-vinylpyridine)	20–120	Drug delivery and pH sensing	2020	(86)
Curcumin, paclitaxel, chlorobenzyladenine	Functionalized polyurethane/polyurea	0.017–0.023	Drug delivery	2016	(88)
2-butyne-1,4-diol, caprylic triglyceride, and jojoba oil	Ketal functionalized polyamide	14	Anticorrosive coatings	2022	(92)
Ibuprofen	Ca/Mg-Alginate	>200	Drug delivery	2019	(99)
2-hydroxy-3-(octanoyloxy)propyl decanoate (ODO), hexyl salicylate, lily oil and lavender oil	Melamine-glutaraldehyde-formaldehyde	19–29	Fragrance retention (laundry)	2022	(114)
Hexylsalicylate	Chitosan-gum Arabic coacervate	35–50	Fragrance retention (laundry and personal care)	2021	(122)
Allyl isothiocyanate	Tannic acid cross-linked gum Arabic-gelatin coacervate	90–254	Flavouring and nutraceuticals	2011	(126)
Neem seed oil	Genipin cross-linked gelatin and carrageenan	50–200	Pesticidal	2010	(133)
Peppermint oil	Transglutaminase cross-linked gelatin/gum Arabic coacervate	10–60	Flavouring	2011	(135)
Jasmine Essential Oil	Transglutaminase cross-linked gelatin/gum Arabic coacervate	0.075–0.38	flavors, fragrances food, textile and pharmaceutical delivery	2014	(137)
Hollow/dioctylsulfosuccinate sodium salt	Poly(methyl methacrylate)	1.2–2.6	Heat insulation	2018	(139)
Methyl Methacrylate	Ag-Alginate	2400–2500	Self-healing/corrosion inhibition in steel	2015	(140)
Chloroform	Poly(dimethyl diallyl ammonium chloride)/ graphene oxide complex and poly(sodium 4- styrenesulfonate)/ Dimethyl dioctadecyl ammonium bromide complex	13–25	Self-healing concrete/corrosion resistance	2019	(142)

The impact of shell thickness not only applies to polymeric capsules but to all capsule materials. Empirically, the active ingredient or internal phase flux (when driven by molecular diffusion) can be described by eq 1 with the proviso that volume *V* (m^3^) of the receptor medium and diffusion coefficient *D* (m^2^·s^−1^) are constant:
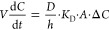
1where *C* is the concentration (mol·m^−3^) in the microcapsule core, *t* is time (s), *K*_D_ is the distribution coefficient (partition coefficient with consideration of ionization state), *h* (m) is the shell thickness, *A* is the exposed capsule surface area (m^2^), and Δ*C* (mol·m^−3^) is the concentration driving force (i.e., the difference between the concentration within the capsule and in the medium).^67^

#### 2.1.1 pH and Temperature Responsive Microcapsules

In some industrial scenarios, the release of active ingredient from capsules may be triggered by a physicochemical change in the surrounding environment. These changes concern most commonly pH, ionic strength, and temperature. The resulting active ingredient delivery may be referred to as a stimulus responsive release mechanism. Ideally, a capsule of this nature should retain the active ingredient indefinitely, until exposed to the relevant stimulus. However, this is rarely the case due to “imperfect” nature of the shell. Both organic and inorganic shell chemistries can result in highly porous networks that would undermine the retainability of the active ingredient over time.

Uncontrolled early release of active ingredient is especially detrimental in a biological or therapeutic setting as an ideal capsule must be able to reach its intended target before active ingredient release is activated at the target site. This often involves navigating the digestive (acidic pH) and intestinal systems (close to neutral pH). A stimulus response is often a function of the polymers or copolymers used to form the microcapsule.^68^ The most common types of triggers are pH and temperature using the polymers polymethacrylic acid (PMAA) and poly(*N*-isopropylacrylamide) (PNIPAM), respectively. These polymers often take the form of hydrogel capsules.^68−70^ They can also be used within the capsule shell to control the active ingredient diffusion from within the capsule to the external medium.

An example concerns PMAA polymer, which may be deposited as a film onto silica particles used as a template may then be removed, leaving behind a hollow polymeric shell.^69,71^ Once the pH is increased above the polymer p*K*_a_, the carboxylic acid groups become charged, leading to swelling of the hydrogel or capsule. As a result, the pore size of the capsule is increased allowing for faster active ingredient diffusion from the capsule into the medium. While impressive in theory, the removal of the silica template requires the use of harsh and dangerous reagents (such as HF) which are unlikely to be implemented on scale in a consumer product. Another example of the use of PMAA that does not require harsh chemical template removal is reported in recent work by Jeon et al., who used a custom microfluidic device to form poly(propylene oxide) (PPO)/PMAA microcapsules (169 μm diameter) from a water-in-oil-in-oil-in water (W_1_/O_1_/O_2_/W_2_) triple emulsion droplet template.^72^ The PMAA acts as a hydrogen bond donor, and the PPO as an acceptor, allowing for interfacial complexation. The permeability of these capsules could be controlled by varying the molecular weight (Mw) of PMAA used in preparation, with decreasing Mw reducing permeability due to a denser polymer network. When between pH 2 and 4.5 there was less than 10% loss over 2 h of the encapsulated pepsin, above pH 5 however, complete release and capsule disintegration took place within 2 h. This is due to the ionization of the PMAA allowing for increased diffusion and disrupting the hydrogen bonding network at the interface.

**Figure 2:**
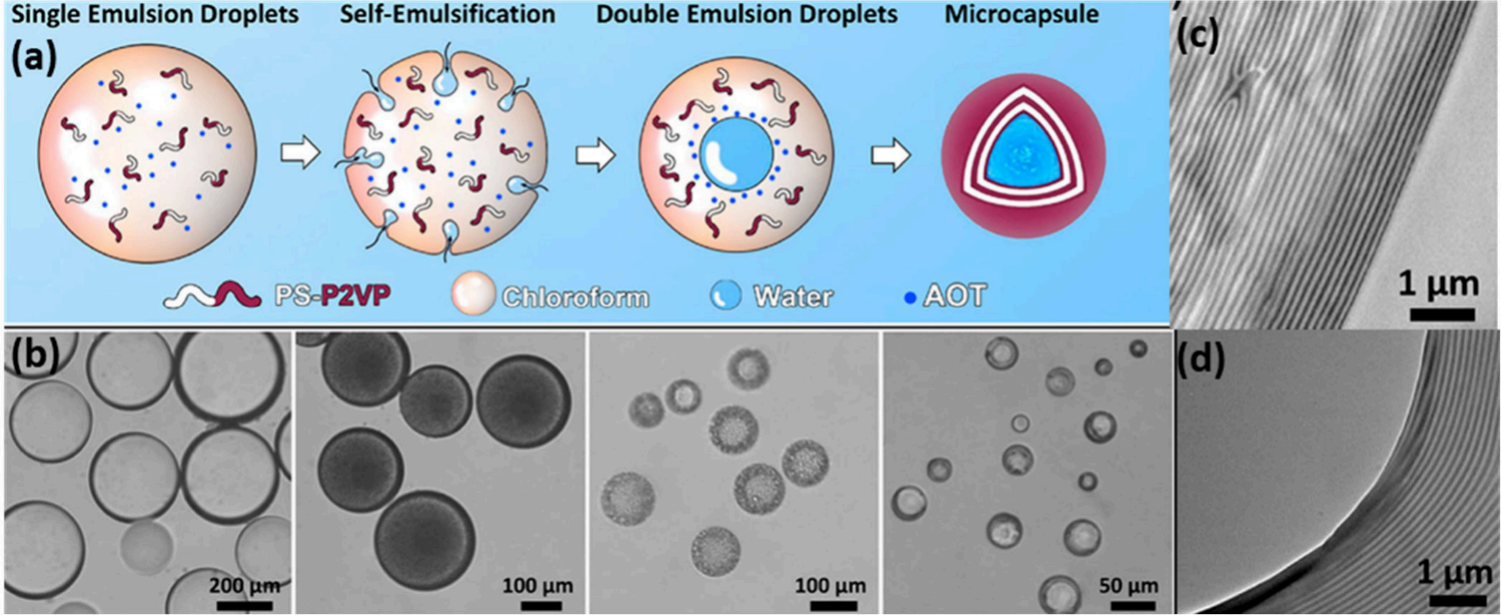
(a) Schematic of the formation of PS-*b*-P2VP PC microcapsules through self-emulsification and solvent evaporation in the presence of poly(vinyl alcohol) (PVA) and bis(2-ethylhexyl) sodium sulfosuccinate (AOT). (b) Corresponding optical micrographs. TEM images of the microcapsule cross section showing the structure of (c) outer and (d) inner surfaces. Adapted with permission from ref (86). Copyright 2020 American Chemical Society.

PNIPAM has been deposited via graft polymerization into the pores of existing polymeric capsules, effectively leading to the formation of temperature responsive gateways.^73,74^ Below the lower critical solution temperature (LCST), PNIPAM polymer chains are swollen and act as a barrier to diffusion. However, above the LCST the chains collapse, effectively opening up a pathway for faster diffusion, this effect may also be further tuned by modifying the polymer grafting density.^75^ This is in contrast to the behavior of PNIPAM as a core–shell encapsulant. In this case, as the temperature rises above the LCST, the capsule collapses inward, causing a decrease in size, and subsequently a significant decrease in active ingredient permeability.^76−79^ PMAA is not the only pH responsive polymer with proposed use in encapsulation. Poly(2-(dimethylamino) ethyl methacrylate (PDMAEMA),^80^ poly(2-vinylpridine), (P2VP)^81^ and chitosan^82^ are all cationic pH responsive polymers that have been reported for potential industrial use without the need for harsh chemical environments, often in combination with other materials such as polydopamine (PDA)^83^ or incorporated via LbL assembly or block copolymerization.^84,85^ One such example is the use of PS-P2VP diblock copolymer reported by Yang et al.^86^ where PS-P2VP was mixed with bis(2-ethylhexyl) sodium sulfate (AOT) in chloroform and emulsified in the presence of poly(vinyl alcohol) (PVA). This resulted in a double emulsion (water–oil–water), once the chloroform evaporated a capsule was formed. This capsule consisted of a lamellae structure which could be tuned by changing the Mw of the constituent monomers (Figure 2). Release properties of these capsules as a function of pH were studied using rhodamine 6G in the internal aqueous phase, which showed that release profiles could be controlled by changing the shell thickness as outlined above or by varying the pH. As pH decreased, the release rate increased, and at pH 7 no more than 50% release after 25 h was reported for the thinnest shell thickness (8 μm). Furthermore, a color change was observed within the capsule as a function of pH and release, demonstrating the dual purpose of these capsules as photonic microcapsules (which are advantageous as diagnostic probes as well as pigments) capable of delivering hydrophilic cargo. It should be noted that the presence of PS blocks will limit biodegradability.

Stimuli responsive polymers are not restricted to a single response. A simple example is the use of a poly(NIPAM-*co*-MAA) polymer shell, which is simultaneously pH and thermoresponsive.^87^ Work by Cuscó and co-workers combined pH and redox responses and developed a multistimulus responsive nano capsule for drug delivery.^88^ Capsules of 16–23 nm diameter were loaded with drugs (paclitaxel and curcumin). These nano capsules were formulated to possess a neutral charge at physiological pH (7.4), reducing electrostatic binding to unwanted residues on cell surfaces. However, when entering the extracellular matrix of a tumor–a traditionally acidic medium,^89^ the capsules become positively charged and were able to bind to and accumulate on the cell’s surface. This allows for these capsules to flow freely throughout the bloodstream until encountering the targeted cancerous site. Furthermore, a disulfide functionality provided by 2-hydroxyethydisulfide allowed for reduction by enzymes and peptides commonly expressed at tumor sites, specifically _L_-glutathione.^90^ On exposure to l-glutathione the capsules demonstrated almost complete release of the internal cargo (fluorescent dye) after 96 h. In addition, when exposed to human serum albumin or bovine serum albumin, little release was demonstrated. These capsules are impressive with regards to their selectivity and potential to avoid off-target effects. However, release studies of untriggered drug capsules were not reported, thus it is unclear if these drugs could be lost to the environment in transit, as potential therapeutics would demonstrate substantially different solubility (and consequently release properties) when compared to the fluorescent dyes. These capsules do however demonstrate a level of biodegradability in vitro and it is likely that this would translate when the capsules are used in real-world applications. It is important to note that the end-use or application of a micro/nanocapsule plays a large role in how complex their formulation can be while being practically viable. Capsules like those described above possessing complex chemistry may not be suitable for high volume/low margins products, however, may be deemed viable for delivery of a chemotherapeutic.

#### 2.1.2 Cross-Linking

**Figure 3:**
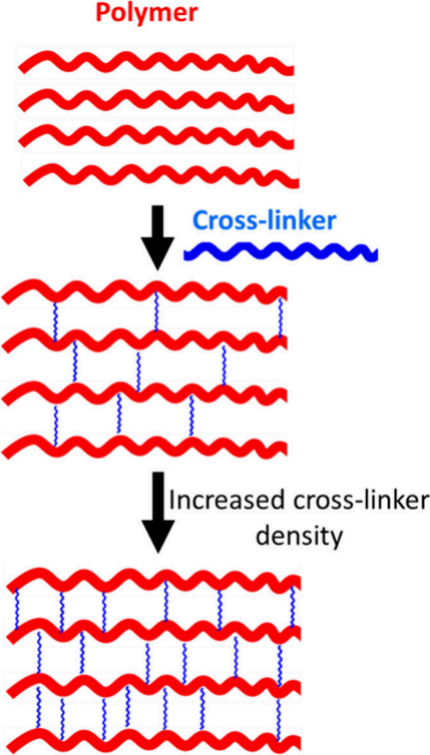
Schematic demonstration of polymer cross-linking and its impact on increasing cross-linker density.

Many examples of responsive polymeric shells use cross-linking agents to tune mechanical and physicochemical properties.^91,92^ One of the most utilized and studied cross-linked systems relates to the polysaccharide alginate family. There are substantial reviews with respect to the versatility of alginate release systems.^93,94^ Briefly, individual polymer chains bearing a negative charge can be bridged or cross-linked upon the introduction of a divalent cation leading to strong intermolecular bonding and formation of a gel. This same principle may be applied to many polymeric systems via a number of different mechanisms including electrostatic, hydrogen bonding and covalent interactions. For more details on these processes, the reader is directed to seminal articles on the matter.^95−98^ Each of these mechanisms results in an increased structural stability of the newly formed polymer matrix. In the case of a microcapsule formed with a cross-linked polymer shell, the cross-linking density can be increased further to decrease permeability, particularly when encapsulating larger active ingredients (Figure 3). For example, recent work by Wang et al. demonstrates this increase in capsule integrity while maintaining a pH response.^27^ Coumarin 1 dye, dissolved in toluene, was encapsulated in a polyampholyte polyamide-based shell cross-linked with triazine, which allowed for a pH-dependent release of the dye. In an oil phase of toluene, the capsules demonstrated no dye release over a period of 5 days. When placed in an aqueous medium, at neutral pH a gradual release was observed, with approximately 40% of the dye being released after 5 days. However, in acidic (pH 5) and basic (pH 10) conditions substantial release was observed, with close to 100% being released within 5 days. The release rate could be modulated by varying the amount of cross-linker, which, as expected, had a significant effect on the shell permeability. As the cross-linker concentration was increased, the permeability decreased while the mechanical stiffness increased significantly, allowing for design tuning. These capsules could be stored either as a dry powder or in organic formulations with almost no loss of the internal phase (dye containing toluene) that composed 95 wt % of the capsule itself. However, the lack of obvious biodegradability may be a hindrance to wide implementation of this technology, which is a pertinent issue that will be described in detail in the subsequent sections. Furthermore, moderate to high degrees of cross-linking typically diminish the degradability of polymer shells as the cross-linking mechanism usually takes place at the same chemical moiety that would be utilized in a biodegradation process.

The type of cross-linker can also play a key role in release rates. For instance, alginate-based hydrogel capsules loaded with ibuprofen prepared by Sanchez-Ballester et al. demonstrated the impact of a number of formulation parameters.^99^ The alginate-based capsules were cross-linked with varying ratios of calcium and magnesium ions. The capsule swelling ratio increased with magnesium salt content at both low and high pH, resulting in an increased release rate of the encapsulated ibuprofen. Similar behavior for a variety of hydrogel systems have been reported throughout the literature.^70,100−103^ It should be noted, however, that irrespective of the magnesium ion content, ibuprofen release (leaching) was below 20% until the pH was raised to 7.2 (simulating the conditions in the intestine), at which point release increased significantly and proportionately to the magnesium ion content. This additional increase in release is due to the ionization of the COOH present on the ibuprofen molecule (p*K*_a_ = 5.2) to COO^–^. In the case of beads cross-linked with calcium only, complete release was achieved after approximately 300 min, whereas complete release was achieved in less than half that time in the case of beads prepared with an Mg:Ca mass ratio of 3:1.^99^ This is because of the weaker interaction of the Mg^2+^ ions with the guluronic acid groups of the alginate polymer, leading to a more porous network. This work demonstrated that different ratios of cross-linking agents may facilitate the environment-induced release of active components from alginate capsules in food, biomedical and pharmaceutical applications.^104^

Although cross-linking agents can provide microcapsules with desirable mechanical and thermo-resistive properties, they can be toxic and environmentally unfriendly. This is reported for formaldehyde, which is widely used in industry.^105−107^ One of the most commonly implemented cross-linked polymeric shells in the laundry industry is melamine-formaldehyde.^108−111^ The cross-linking is well established, however the full reaction pathways to the cured resins, including their chemical intermediates, are poorly understood, and remain under investigation.^109,112^ As a result of the health concerns around the use of formaldehyde, there is research toward safer alternatives.^113^ For example, Luo et al.^114^ have reduced the amount of formaldehyde required to cross-link melamine-based microcapsules laden with lily and lavender oils, and hexyl salicylate. This was accomplished by using glutaraldehyde as a co-crosslinker, thereby reducing the residual formaldehyde content by approximately a factor of 10 (from ∼235 ppm to ∼22 ppm), without affecting the mechanical properties of the resulting microcapsules, which remain MP based. When compared to formaldehyde (HCHO), the biological acceptance of glutaraldehyde appears to be greater. It is used as a cold sterilizing agent for heat-sensitive medical, surgical and dental equipment albeit large amounts may trigger respiratory sensitization.^115^ Glutaraldehyde ((CH_2_)_3_(CHO)_2_) is a significantly larger molecule than formaldehyde, hence its diffusion through (human) tissue may be significantly impaired. It is used in vivo for pulpotomies, pulpectomies, tooth root canal and intracanal treatments.^116^ However, its toxicokinetic profile is somewhat controversial because the available human/animal studies have pinpointed several potential health outcomes, including respiratory, gastrointestinal, renal, dermal, and ophthalmological effects.^117,118^

Glutaraldehyde reacts with free amines located along a proteinaceous backbone of biopolymers, triggering irreversible covalent bonds.^119^ For this reason, glutaraldehyde has been employed in the textile industry as a finishing agent for cotton and wool fabrics.^120^ It has proven effective at cross-linking and strengthening perfume microcapsules for laundry formulations,^13^ which can be exposed to challenging conditions of pH/temperature during washing-drying cycles.^121^ To this end, Baiocco and co-workers^122^ have explored the feasibility of fabricating glutaraldehyde cross-linked microcapsules laden with perfume oil, within a MP-free shell made of plant-based chitosan and gum Arabic by coacervation, with potential applications in laundry formulations. Although the physiological toxicity of glutaraldehyde is dose-related, human exposure must never be direct,^121^ which precludes its utilization in many everyday products.^123^ Therefore, the scientific community is proactively shifting to identify safe, cost-effective and universally suitable cross-linking alternatives for food, pharmaceutical, and cosmetics microencapsulation.^124^

Sodium tripolyphosphate (NaTPP),^125^ glyceraldehyde,^123^ tannic acid,^119,126^ polyphenols,^127^ genipin,^128^ and transglutaminase enzyme (TG)^119,129^ have drawn interest. Specifically, multivalent anionic NaTPP (P_3_O_10_^5–^) was employed on gum Arabic and animal chitosan microcapsules, which can interact electrostatically with chitosan’s positively charged amines (NH_3_^+^), to form ionically driven intermolecular linkages, and hence cross-linked networks.^124^ A subsequent study, which used NaTPP to cross-link an aqueous chitosan solution in oil emulsion to form particles, demonstrated a reduction in adhesion of the chitosan surface due to surface charge neutralization.^130^ Similarly, tannic acid has been used to cross-link gelatin–gum Arabic coacervate microparticles for the controlled release of food flavouring allyl isothiocyanate.^126^ Alternatively, genipin is a naturally occurring, nontoxic and biocompatible cross-linking agent.^131,132^ Microcapsules made from gelatin type A and κ-carrageenan were fabricated for the encapsulation of antioxidant, neem oil. The capsules were cross-linked with genipin. As discussed above for pharmaceuticals, it was also found that the release rates of the active was dependent on the amount of cross-linker.^133^ Likely, genipin reacted spontaneously with amino acids along the gelatin’s backbone to provide cross-linking. Unlike glutaraldehyde, genipin is capable of binding to only one other genipin molecule, hence its cross-linking efficacy may be significantly diminished.^131^

Enzymatic cross-linking represents another interesting alternative in food formulations and microencapsulation processes. Specifically, naturally occurring transglutaminase (TG) has proven suitable for covalent protein binding, which leads to the formation of intra- and intermolecular ε-(γ-glutamyl) lysine bonds.^134^ Dong et al.^135^ achieved thermally resistant (release rates were not substantially affected by temperature) microcapsules with reduced active leakage using TG. In contrast, Grosso and co-workers^136^ and Lv et al.^137^ argued that the performance (barrier properties) of TG-cross-linked microcapsules are not as robust as glutaraldehyde cross-linked microcapsules. To date, although several safe and eco-friendly cross-linking alternatives have arisen, they have not been widely implemented at an industrial scale, possibly due to processing/material costs and the overall performance of the resulting microcapsules.^67^ However, due to impending regulations outlined below, this is a rapidly evolving field and major manufacturers have begun to take such capsules to market. For example, laundry microcapsules are utilized for prolonging the fresh and clean scent on fabrics. Therefore, it is pivotal for microcapsules containing volatile perfumes to remain undamaged and impermeable (i.e., no oil leakage) until a desired time, at which point they can be broken mechanically after the washing/drying cycles, releasing the scented active. Achieving this objective has been accomplished primarily via formaldehyde and glutaraldehyde-formaldehyde cross-linking techniques, which have a proven remarkable efficacy.^61,114,138^ For example, the aforementioned melamine formaldehyde capsules have been used as an industry standard due to their ease of manufacture, water and heat resistance, hardness and smooth morphology, owing to the crystalline nature of the melamine. Once cross-linked with formaldehyde, this allows for the production of a very low porosity material.

Capsules may act as standalone delivery devices but can also be imbedded in films or layers.^139−141^ Work by Thakare et al. reported capsules embedded in an epoxy coating spread upon a steel substrate.^92^ These microcapsules were formed via an interfacial polymerization/cross-linking process and were designed to encapsulate two different anticorrosive active ingredients (2-butyne-1,4-diol and jojoba oil). Cross-linking was accomplished through covalent bonding between amine groups on diethylaminoketal and acyl chloride moieties of trimesoyl chloride. This resulted in a polyamide shell, which was modified with ketal functionality to induce a pH response in acidic conditions (prevalent in corrosive environments). At pH 7 and 9 the capsules demonstrated no release (up to 8 h). Once the pH was lowered to 5, a gradual release was observed with 100% release occurring within 2 h. These capsules showed between 20% and 70% corrosion inhibition depending on the pH, type of encapsulated active and its concentration. These capsules could also be stored for up to 3 months in nonacidic conditions with no observed release or reduced function. Furthermore, the microcapsules demonstrated no significant adverse effects on the adhesion of the epoxy coating on the steel substrate at 10 wt % loading. It should be noted, however, that similar anticorrosive coatings operate at between 5 and 30 wt %. This work not only demonstrates an interesting microcapsule technology, but that these capsules can be placed within other technologies such as paints or other protective coatings and lay dormant until needed to perform their function. These particular capsules are not biodegradeable; by their very nature they are engineered to counter or inhibit natural processes. As a result, if they were readily degradable it would be detrimental to their intended use, an example of the constant compromises having to be considered by formulation scientists in both academia and industry. Cognisant of the developments presented above and increasing global demand, it has become imperative to move toward novel harmless, effective, MP- and animal-free biopolymer-cross-linking pairs. These designs are actively pursued in both academic and industrial spheres.

### 2.2 Triggered Release Capsules

Triggered or burst release refers to a release profile that does not begin until triggered by an external stimulus such as the polyamide shell outlined in Table 1. The advantages of burst release are that the active is isolated from the surrounding environment until a condition change activates its release (Table 2). However, it is paramount that no leaching of the active ingredient is achieved when the capsules are “dormant”, to obtain the most effective performance. This results in a more efficient product delivery to the target, thus potentially allowing for a lower dose requirement. Optimization of the available microencapsulation technologies has gained increased attention toward overcoming the shortcomings associated with leaching. While many different types of polymeric capsule formulations have been reported in the literature, such capsules are typically not suitable for the encapsulation of low molecular weight active ingredients for long durations due to the inherent porosity and permeability of the polymer shell as outlined above.^30^ However, when a polymeric shell is heavily cross-linked, its permeability can be significantly reduced, possibly leading to a longer-lasting retention of the active ingredients. Nevertheless, heavily cross-linked shells often result in microcapsules that are largely nonbiodegradable. An additional method used to mitigate unwanted leaching is the use of capsule shells that are chemically incompatible with the active ingredient. This approach relies on the reduced partitioning of the active (and hence reduced diffusion through the shell) to entrap the active in the dispersed phase. One example is reported by Ziering et al., who used microfluidics to form a double emulsion. This double emulsion was then used as a template for photopolymerization of a cross-linked perfluoropolyether, a lipophobic and hydrophobic polymer.^143^ These capsules could be formed with either an aqueous internal phase, CaCl_2_ and Allura Red solution, or an organic toluene and Nile Red phase. When subjected to a glucose environment the aqueous core capsules exhibited little release, with the osmotic pressure being relatively small. However, when placed in pure DI water the high osmotic pressure, caused the capsules to begin to release their core, reaching 14% release over 25 days. Conversely, when the oil-core capsules were placed in toluene, their release was rapid, releasing 80% of the encapsulated dye instantaneously.

**Table 2:** Summary of Triggered Release Capsules Presented in Section 2.2

core	shell	size (μm)	proposed application	year	reference
Toluene-di-isocyanate	Composite shell consisting of graphite, paraffin and polyethylene wax	10.7, 105 and 800	Self-healing concrete	2021	(24)
Allura Red (CaCl_2_ solution) Nile Red (toluene)	perfluoropolyether	160	Agriculture, cosmetics, and drug delivery	2015	(143)
Paclitaxel	Gold	10	Medical: delivery of cytotoxic drugs	2020	(144)
Hexyl salicylate	Calcium phosphate	1–5	Food/Pharmaceutical	2022	(145)
Kanamycin	Silver/Gold	0.9–3.2	Medical: delivery of antibiotics	2018	(146)
		0.8–2.1			
Doxorubicin	Silver	0.9–3.2	Medical: delivery of anticancer drugs	2017	(147)
Doxorubicin	Gold	0.8–2.1	Medical: delivery of anticancer drugs	2018	(148)
Hexadecane	Gold	2- 25	Medical: delivery of cytotoxic drugs	2019	(149)
Miglyol 812	Gold	12.5	Medical: drug delivery	2019	(150)
Polyacrylic acid	Silica	29.5	Medical: self-healing of cracks in dental composites	2016	(158)
Linseed oil	Urea-formaldehyde resin	5–100	Healing cracks in paint/coatings	2008	(159)
Oil soluble solvents and reactive epoxy resins	Urea-formaldehyde	10–300	Self-healing of epoxy films	2009	(160)

**Figure 4:**
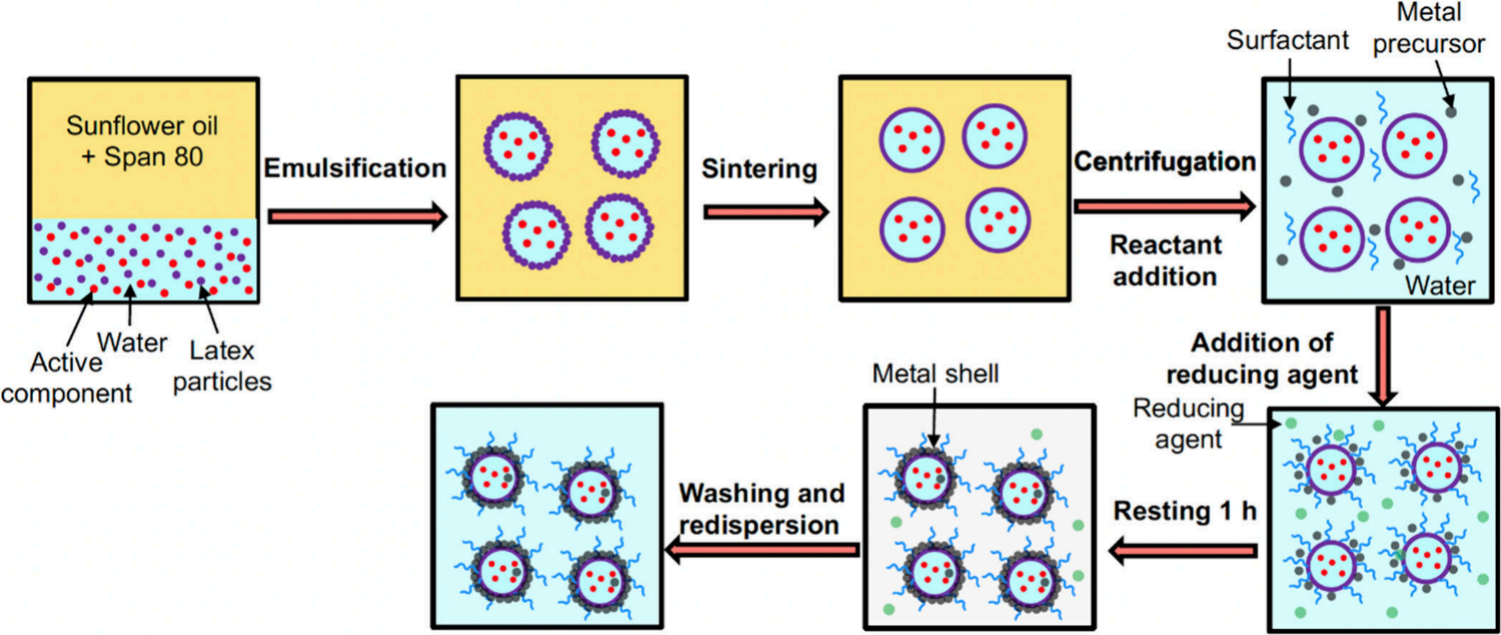
Schematic of colloidosome template for metal shell encapsulation of an active material, such as a pharmaceutical. Here, a water–oil emulsion is first formed where the internal water phase contains the active ingredient. Once, the particles adsorbed at the interface are sintered and the continuous oil phase is replaced with water, a metal precursor is used to reduce a metal film on the surface of the colloidosome structures.

Overall, when active ingredients are successfully encapsulated within a “liquid-tight” shell, a burst release is typically obtained only through mechanical stimulation. Subjecting the capsules to high mechanical stress, such as rubbing and scratching, can lead to cracks propagating through the shell surface. This leads to burst release of the active.^138^ This method of release is not limited to polymeric materials and many inorganic shells have been produced which follow a similar mechanism.^30,144−147^ For example, a number of studies have aimed to prepare metal-coated microcapsules that are capable of fully retaining the encapsulated material for weeks or months,^30,144,146−150^ which is then released by an external trigger at the desired location. These metal-shell capsules come in a variety of forms with both aqueous and oil cores, utilizing both polymeric and inorganic substrates for metallic film growth.^30,144,146,151,152^ One such example is metal-coated colloidosomes, which consist of an aqueous core with the active component, e.g., drug, and a polymer shell that is covered with a second metallic shell preventing the diffusion of the encapsulated material. Figure 4 shows the generalized steps of preparing metal-coated colloidosomes with an aqueous core containing the active, and an intermediate polymeric shell.

#### 2.2.1 Inorganic Shells for Drug Delivery

**Figure 5:**
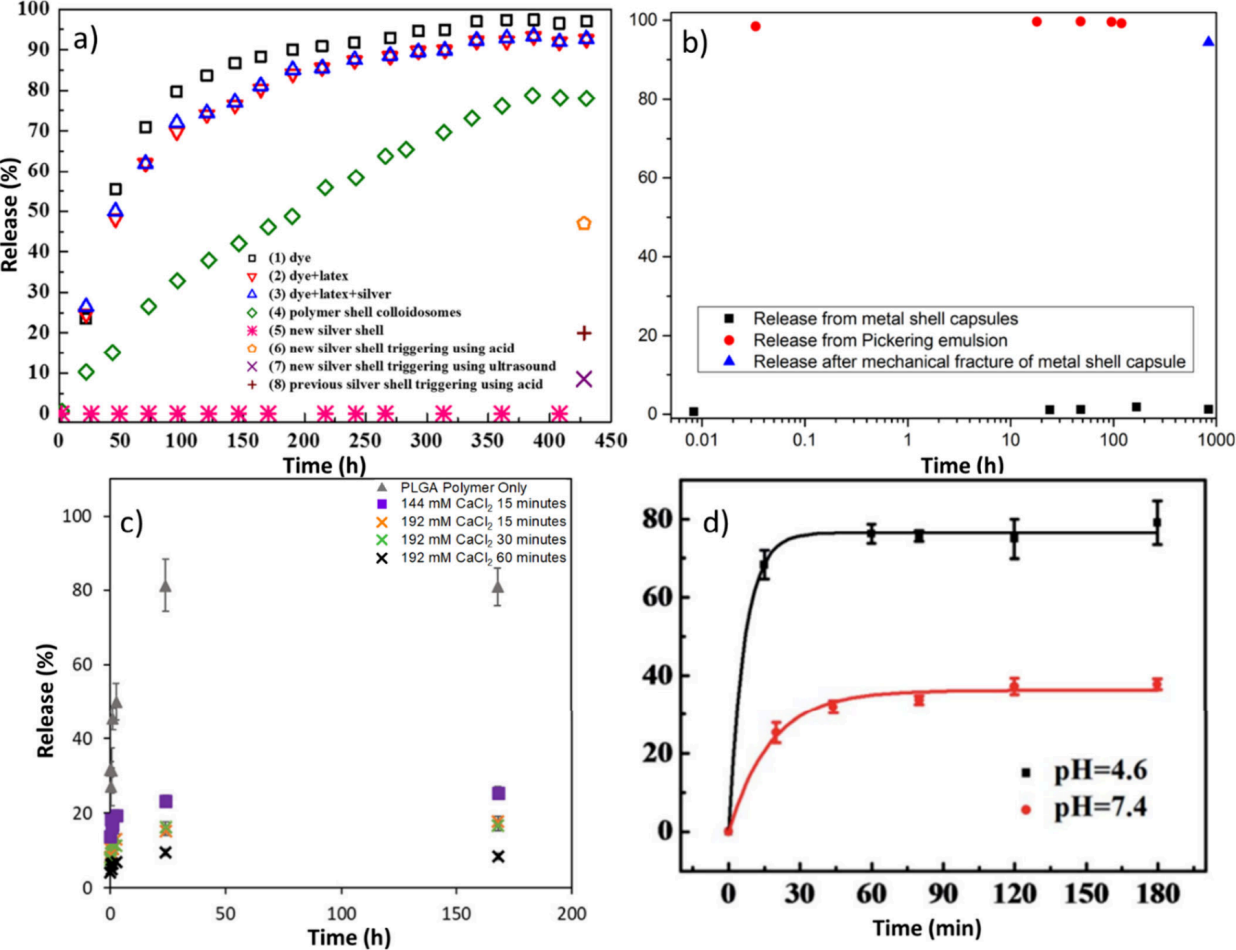
Release profiles of inorganic shell microcapsules. (a) Release profile of Allura Red dye contained within silver-coated colloidosomes at various stages of composition and using both acid and ultrasound triggers into water. Modified with permission from ref (147). Copyright 2017 American Chemical Society. (b) Release profile of gold-plated emulsion droplets and bare emulsion droplets with and without mechanical fracture into a 4:1 (v/v) ethanol/water medium. Modified with permission from ref (149). Copyright 2019 American Chemical Society. (c) Release profile of hexyl salicylate into ethanol before and after calcium phosphate coating at different concentration and coating times at 55 °C. Modified with permission from ref (145). Copyright 2022 Elsevier. (d) Release rate of calcium phosphate capsules coated in ferric tannate. Reproduced with permission from ref (155). Copyright 2015 Royal Society of Chemistry.

An example of metal-shell capsules is reported by Sun and co-workers who developed impermeable silver-coated colloidosomes for the encapsulation of doxorubicin, a low molecular weight anticancer drug. The capsules had a spherical shape with a size between 0.9 and 3.2 μm. The silver-coated colloidosomes were triggered by ultrasound to break the silver shell and release the drug. A release study was performed on silver-coated microcapsules containing Allura Red AC, which was selected as a model for the release as it does not impact the production of colloidosomes and is small enough for diffusion through the pores of the polymeric shells. The ultrasound treatment of the microcapsules resulted in a low release yield of around 8.5%, as only the microcapsules near the ultrasonic probe were broken (Figure 5a). Cells were killed by the released doxorubicin and the fragments of the silver shell. The impermeable and nontoxic nature of the silver shell makes microcapsules potentially suitable for biological and medical applications as carriers of small hydrophilic drugs.^147^ Later, this work was expanded by the same group who produced a gold shell using a similar methodology for encapsulating drugs and antibiotics.^146,148^ For example, Sun et al. developed impermeable colloidosomes that had an aqueous core and an intermediary polymer shell, surrounded by a second metallic gold shell for the encapsulation of anticancer drugs. The gold-coated microcapsules had a spherical shape with a diameter ranging from around 0.8 to 2.1 μm. The release of the anticancer drug, doxorubicin, was successfully triggered using ultrasound. The released drug in combination with the broken shell fragments killed B50 cancer cells. It was demonstrated that no release was observed from the gold shell capsules containing Allura Red AC, compared to the polymer shell capsules, which exhibited a maximum release yield of 83.7% after 900 h. The gold-coated capsules ruptured by ultrasound treatment again showed a low maximum release yield of around 8.4%. The low toxicity, complete retention of the core material as well as the capability of triggering the release by ultrasound at the desired time and location, make such capsules suitable as carriers for anticancer drugs in the medical field.^148^ Furthermore, it is likely that this release could be improved or optimized by varying the ultrasound power or waveform (frequency and cycle interval).^153^

Using the same encapsulation technique, Sun et al. were also able to develop impermeable silver or gold-coated colloidosomes containing an antibiotic, kanamycin. The release of the active component was triggered using ultrasound to break the metal shell. Such capsules can be used in biological systems to kill bacteria. The study shows that the released antibiotic in combination with the broken metal fragments, killed *Escherichia coli* (*E. coli*). Loading kanamycin did not have a significant impact on the morphology of the gold shell and therefore the capsules retained their spherical shape. In contrast, the silver-coated capsules loaded with kanamycin had a different shell morphology compared to the water core equivalents as the shell was composed of thinner silver particles with some silver sheets becoming hollow at the edge. This might be attributed to the interaction between the kanamycin and silver precursor (i.e., kanamycin impacted the reduction of silver ions into metallic silver), which in turn impacted the morphology of the silver shell and hindered the formation of shell-shaped capsules.^146^ This shows that careful selection of the core and shell materials is important to prepare the desired metal-coated capsules. Besides the desired properties, such as the biocompatibility of the polymeric and metallic shells and the full retention of the core active ingredient until its release by an external trigger at the desired location, the intermediate polymeric shell of such colloidosomes was composed of synthetic nonbiodegradable latex particles, i.e., poly(methyl methacrylate-*co*-butyl acrylate), which represents a drawback to their usability or practicality in certain applications.

This type of metal-shell capsule is not limited to colloidosomes but can also be produced using a traditional oil/water (Pickering) emulsion as a capsule template.^40^ Stark et al. synthesized microcapsules of diameter 2–25 μm with an impermeable gold shell for the encapsulation of an oil core using a two-step method: stabilizing an oil-in-water emulsion with catalytic platinum nanoparticles (Pickering emulsion) followed by an electroless deposition process. The continuous thin gold film deposited onto the emulsion droplets ensured complete retention of the oil in a continuous phase that completely dissolves the microcapsule oil core, i.e., a 4:1 mixture of ethanol and water. Complete retention was observed even after 41 days (Figure 5b). This method is simple, scalable, has a high loading of active component and enables the production with controlled properties, such as diameter and density of the microcapsule as well as the thickness of the secondary metal film. Such microcapsules can be remotely fractured using ultrasound making them candidates for the delivery of cytotoxic drugs.^149^ The potential of using ultrasound as a release mechanism was investigated by White and co-workers who explored the response of these nonpermeable gold-coated microcapsules with and without an intermediate polymer shell. The microcapsules were spherical with an average diameter of 12.5 μm. The work investigated the use of focused ultrasound (FUS) for rupturing the microcapsules. It was found that the gold-coated microcapsules with an intermediate polymer shell were successfully fractured using FUS, corresponding to acoustic pressures as low as 0.5 mPa (∼3.2 × 10^5^ (J·m^−3^)·s^–1^) of the surrounding liquid. In contrast, the microcapsules without the intermediary polymer shell exhibited a different release behavior in other bulk phases. The response of the microcapsules to FUS decreased in an aqueous solution, whereas embedding the microcapsules in a hydrogel matrix resulted in full release of the encapsulated material between 7 and 35 days.^150^ This is because the capsules were trapped in the gel and subjected to a stronger FUS force. It is worth mentioning that the metal capsules were prepared with and without intermediary shell polymers, which can be biodegradable, such as polylactic-*co*-glycolic acid copolymer (PLGA). Furthermore, Hitchcock et al. were able to demonstrate the encapsulation of paclitaxel within these impermeable gold shells and release the drug on demand. However, it appeared as if some of the drug was retained on the gold shell itself and could not be recovered after rupture.^144^ This work demonstrated control of the capsule size, bulk density, and morphology through tuning of constituent concentrations. These outcomes were further verified in a recent subsequent publication by Stark and co-workers.^154^

The use of metals, such as gold or silver, as a shell material for microcapsules can be too expensive for many applications and therefore investigating alternative inorganic shell materials, such as minerals, that are capable of achieving full retention of small molecules is crucial. White and coauthors developed a novel method to deposit a continuous impermeable thin mineral shell, such as calcium phosphate, onto polymer microcapsules with a liquid core.^145^ Such microcapsules are capable of providing efficient encapsulation and protection of small molecules. This was achieved by using platinum nanoparticles as a catalyst to induce a direct nucleation and growth of the calcium phosphate shell. The uncoated polymer microcapsules had a spherical shape with a size ranging between 1 and 5 μm and a biodegradable PLGA shell. Interestingly, the calcium phosphate shell thickness of these microcapsules was determined to be affected by the concentration of calcium chloride, with 96 mM and 192 mM leading to 40 or 60 nm, respectively. It was found that the majority of the microcapsules had a nonporous shell with low release of the encapsulated core component. Release occurred only from the microcapsules with defects over a period of 7 days or upon triggering the release of the encapsulated core oil, i.e. hexyl salicylate, using an organic solvent, i.e., ethanol (Figure 5c). Calcium phosphate is cheap, biodegradable and biocompatible, which makes it a good candidate as a shell material for applications where the biocompatibility and protection of active ingredients are required, such as oral delivery of drugs and nutritional substances.^145^ Further studies are required to determine whether a pharmaceutical active can be delivered in these capsules and if rupture on demand can be achieved given the notorious hardness of CaPO_4_ based materials.^156^ These studies are key to determine how effectively these materials can be implemented in drug delivery systems. Without being able to accomplish a triggered release, careful optimization of the shell thickness would have to be undertaken for each individual active to be encapsulated, so as to allow for controlled diffusion through the inorganic shell. This impressive retention is in contrast to calcium phosphate shells reported by Su et al, who formed calcium phosphate microcapsules via interfacial crystallization during ethanol/water mixing. The capsules were produced by saturating an ethanol solution with K_2_HPO_4_ and mixing with CaI_2_ solution and additional water, which led to mineralization and formation of microcapsules.^155^ While these capsules exhibited some pH responsive behavior, their overall retention was not as impressive as those reported in ref^145^ as at pH 4.6, almost 80% of the encapsulated material was released within 20 min, and at pH 7.6, 30% released within 30 min (Figure 5d). Long-term retention studies were not reported.

#### 2.2.2 Microcapsules for Structural Augmentation

**Figure 6:**
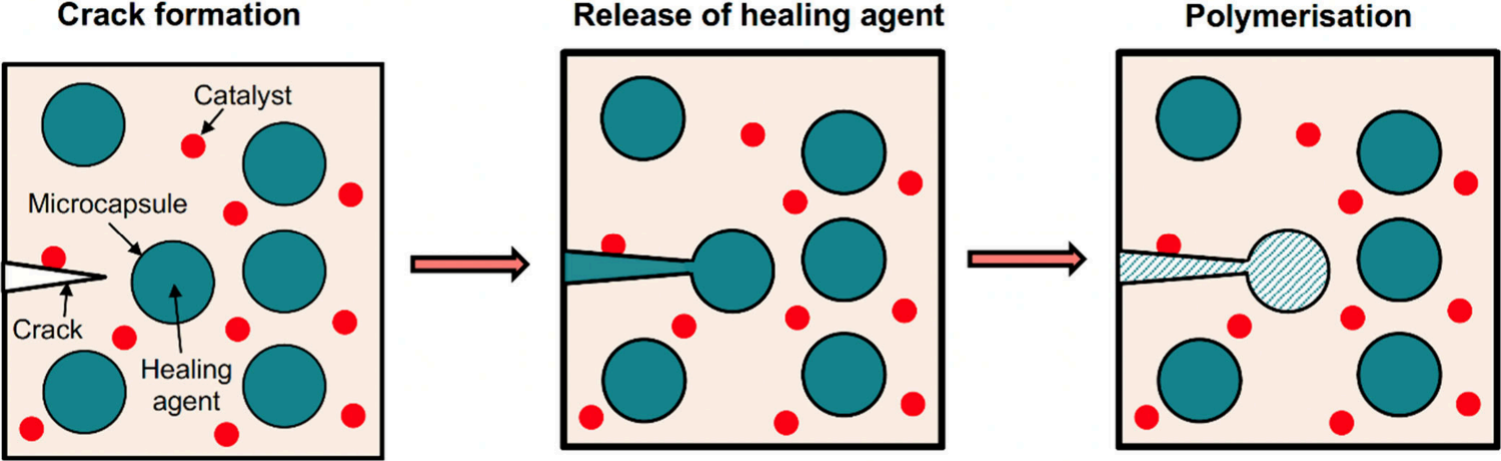
Concept of self-healing of cracks using microcapsules. The self-healing process involves the rupture of microcapsules containing a healing agent due to crack formation, release of the healing agent from the ruptured microcapsules into the formed crack, and filling the crack with the polymerized healing agent.

Burst release capsules are not confined to drug delivery applications. There has been a recent increase in reports using microcapsules as tools to automatically heal cracking in both biological and mechanical structures. The self-healing of cracks is a promising approach underpinned by the ability of microcapsules to retain the healing agent in their core and release it upon triggering. Figure 6 shows the concept of self-healing using microcapsules to repair damage caused by the formation and propagation of cracks over time. A monomer, i.e. self-healing agent, is encapsulated in microcapsules, which are then embedded within the matrix of the bulk material. The development of a crack results in the rupture of microcapsules that are in contact with the crack, which leads to the release of the healing liquid into the formed crack. The healing liquid polymerizes after its release and repairs the crack.^157^

Huyang and co-workers synthesized silica microcapsules containing an aqueous solution of poly(acrylic acid) , via a silica condensation method. The capsules were intended for self-healing of cracks in dental composites. The elastic modulus of silica microcapsules matches that of the fillers in the dental composites, which prevents their premature fracture during production. The spherical microcapsules of an average diameter of 29.5 μm released the encapsulated healing liquid core upon development of microcracks in the resin in which they were embedded. The released core content reacted with the strontium fluoroaluminosilicate particles incorporated into the resin, to form an insoluble reaction product that filled and sealed the generated cracks.^158^

Suryanarayana et al. synthesized microcapsules consisting of linseed oil as the core material and urea-formaldehyde resin as the shell by *in situ* shell polymerization. The capsules were intended for healing cracks in paint/coatings. The 5 to 100 μm microcapsules were ruptured under simulated mechanical action to release the linseed oil and healed the cracks in a paint film through formation of a continuous film in the crack upon the drying of the linseed oil by oxidation with atmospheric oxygen.^159^

Blaiszik et al. reported the use of microcapsules of varying composition to be implemented in the self-healing of epoxy films. They prepared microcapsules composed of an oil soluble solvent and reactive epoxy resin as the core material and a thin, polymeric, urea-formaldehyde (UF) shell. The microcapsules were produced via an *in situ* polymerization of urea and formaldehyde. The shell wall of the microcapsules consisted of two distinct layers: a thin continuous inner shell wall of 160 nm and a thicker rough exterior shell wall. The continuous layer is formed during the UF reaction in the aqueous phase, which results in the deposition of a low molecular weight polymer at the water–oil interface. Upon the progression of the UF reaction, the rough layer is formed due to the coalescence of the UF particles and their deposition along the interface. The 10 to 300 μm microcapsules released their core through rupture of their shell induced by a propagating crack and delivered the reactive epoxy resin to the damaged region.^160^

The capsules discussed so far, offer potential solutions to problems on the micro and milli scale. Microcapsules can also be used to augment bulk materials, such as concrete structures, through the self-healing of mechanical cracks that are formed in such structures over time. Cracking is a common problem in concrete structures that occurs as a result of their intrinsic porosity, as well as exposure to various environmental conditions. Materials, such as self-healing concrete, have been developed to overcome cracking problems. Crack repair is achieved by the addition of various materials, such as microcapsules. Li et al. developed microcapsules for self-healing concrete that were ruptured using microwaves as a trigger. Microcapsules contained a healing agent, toluene-di-isocyanate, and a composite shell that consisted of graphite, paraffin and polyethylene wax. The microcapsules were spherical with a volume-based average particle size of 10.7 μm, 105 and 800 μm.^24^ Although the microcapsules used for self-healing of cracks are capable of retaining the core active material until its release by the crack propagation through the shell, some shell materials of these microcapsules did not appear to be readily biodegradable based on the properties of such materials.

## 3 Energy of Production

**Figure 7:**
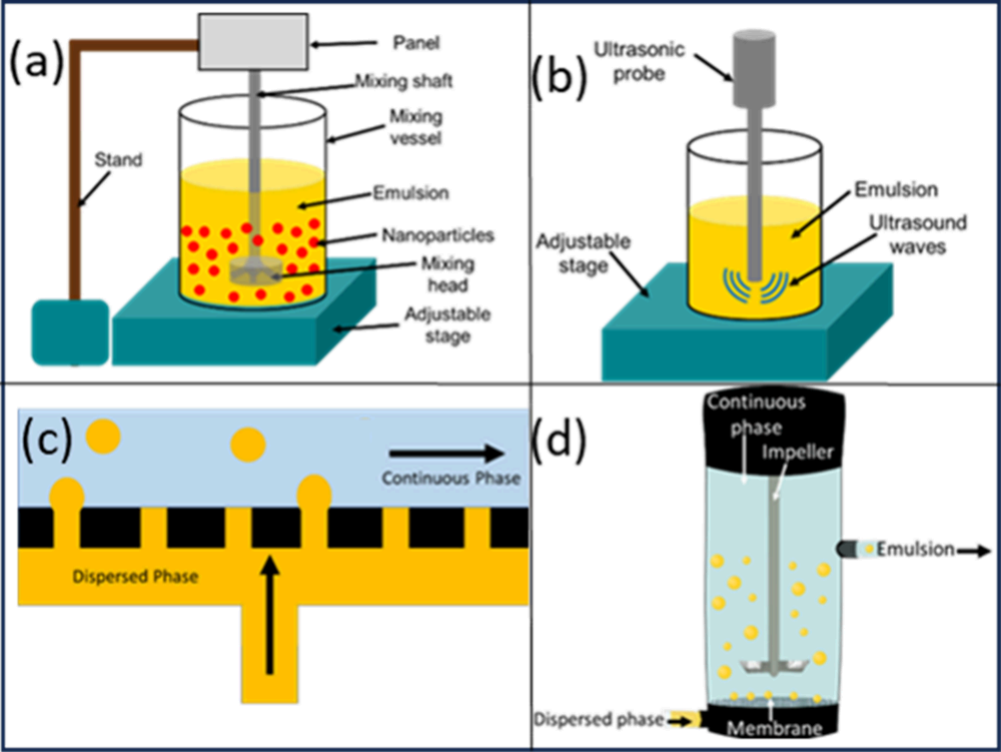
Schematics of general process of emulsification methods. (a) Emulsification using shear mixing, (b) emulsification using ultrasound and (c) crossflow membrane emulsification, (d) stirred-cell membrane emulsification. Images presented do not necessarily represent industrial-scale methods; however, fundamental properties/mechanisms remain the same.

In the sections above, we have focused on the effectiveness and formation mechanisms of capsules found throughout the literature. Aside from these factors, for a capsule technology to be commercially viable, the production costs, both with respect to materials and energy, should be evaluated. In this section we consider some of the main methods of emulsion formulation as a precursor to microcapsule production.^161,162^ Specifically, we discuss mechanical homogenization, ultrasonication and membrane emulsification (ME) (Figure 7). For example, in the food and allied industries, emulsified formulations are typically achieved via high shear mechanical agitation to a liquid admixture using a rotor-stator device such as a colloid mill, a high-pressure membrane or an ultrasonic homogenizer.^163^

### 3.1 Mechanical Mixing

Mechanical mixing is a commonly used method for the preparation of a wide range of emulsion templates. Such templates have been successfully used for the preparation of microcapsules that can be used in a number of applications including the encapsulation of anticancer drugs^147,148^ and antibiotics.^146^ These templates have also been used for the production of composite beads, such as magnetic photocatalytic microbeads for the degradation of dyes.^164,165^ The size of the microcapsules/microbeads is directly related to the energy input of the mixer, i.e. high energy input via an increased mixing speed results in the production of capsules/beads with smaller sizes.^166^ A schematic of a typical shear mixer is illustrated in Figure 7a.

It has been customary to correlate the Sauter mean drop size (*d*_3,2_) to the Weber number, (*We* = drag forces/cohesion forces or, in other words, equals the ratio of the kinetic energy on impact to the surface energy) in order to estimate the typical energy generated in an agitated vessel.^162^ In 1958, Calderbank first described the influence of the surface tension on the resultant interfacial area by assessing the effect of a number of aqueous hydrocarbon dispersions at a relatively large scale (<0.1 m^3^).^161^ Their model can predict an increase in the interfacial area with a decrease in the surface tension (σ,N/m) i.e. *area* ∝ σ^6/5^:
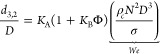
2where *D* (m) is the impeller diameter, *K*_A_ and *K*_B_ are dimensionless proportionality constants, Φ (dimensionless) is the volume fraction of dispersed phase, ρ_C_ is the density of the continuous phase, and *N* is the stirring rate (s^–1^). For a nonviscous dispersed phase within a stirred tank equipped with a six-blade Rushton turbine *K*_A_ and *K*_B_ were experimentally determined to be 3/50 and 9, respectively. Interestingly, for nonideal and concentrated systems where coalescence phenomena become significant, *K*_B_ can be much higher (e.g., ∼ 22 for chlorobenzene in water), hence indicating, as expected, a tendency of the dispersed droplets to coalesce promptly.^162^ With *d*_3,2_ being predicted via (2), it is therefore possible to estimate the minimum energy (J·m^−3^) required to produce an emulsion of a particular average diameter:
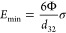
3

High shear mixers (HSMs) are widely utilized in several industries, such as the chemical, pharmaceutical, food, cosmetics and paint industries for the production of emulsions with narrow droplet size distributions and to form emulsions with small droplet sizes and large interfacial areas.^167^ Most of the energy used for the preparation of emulsions by high shear mixers is consumed during the mixing process, and therefore it is crucial to estimate the energy of mixing. An empirical correlation between the Sauter mean diameter, *d*_3,2_ (m), and the energy density, *E*_V_ (J·m^–3^) is given by the following equation,
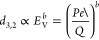
4where *Pe* is the total power input (W) and *Q* is the volumetric flow rate (m^3^·s^–1^).^167^

### 3.2 Ultrasound in the Formation of Emulsion Templates for Microcapsules and Their Release

Ultrasonically assisted methodologies (UAMs) are popular in many industrial fields including biomedical diagnostics (e.g., 2D, 3D, and 4D scan of fetuses), cleaning in place, and forming, welding, cutting and inspecting steels or plastics against cracks and inhomogeneities.^168^ Generally, UAMs entail short pulse soundwaves (typical frequency 0.025–20 MHz) which are not perceivable by humans whose absolute acoustic spectrum ranges between 20 Hz and 20 kHz.^169^ Ultrasounds emit longitudinal soundwave pulses at a frequency above 20 kHz. The velocity of sound (c) heavily depends on the nature of the medium the soundwave is moving through. Therefore, the corresponding wavelength (λ) is given by,
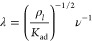
5

Where ν is the frequency (s^–1^), and  and 1/*K*_ad_ are the density (kg·m^–3^) and the adiabatic compressibility of the liquid medium (m^2^·N^–1^) respectively. Clearly, the energy transferred into the liquids to be emulsified via UAM is linked to the intensity (i) of the sound wave per unit time (J·m^–2^ s^–1^)
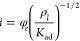
6that is, in turn, related with the energy density φ_e_ (J·m^–3^):
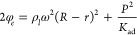
7which is given by two contributions, namely the kinetic and potential energy densities; where R and r are the geometrical radii of the reactor and impeller, respectively, **ω** is the rotational speed of the impeller (or velocity of the particles), and *P* is the soundwave’s pressure amplitude defined as the crest-trough pressure difference (N·m^–2^). Consequently, the volumetric wattage W, (W·m^–3^) over the required batch time Δt (s) (e.g., encapsulation, release, extraction) is simply calculated as follows:
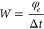
8

**Figure 8:**
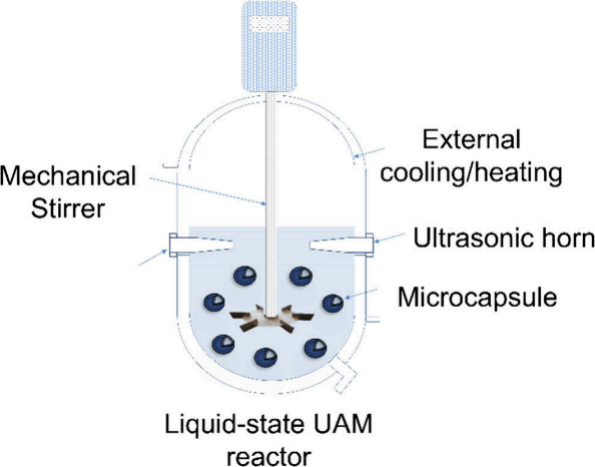
Diagram of an experimental setup for ultrasonically assisted stirred reactors for microencapsulation, extraction, and release processes.

Based on the above, the schematic of a UAM (bio)reactor with an industrial-like configuration is depicted in Figure 8. Such a setup could be used for ultrasonically assisted microencapsulation, and release/extraction of the microcapsule actives.^170^ This setup requires a jacketed vessel equipped with a stirring turbine and an ultrasonic horn. Cavitation is desirable in many sonochemical processes, therefore the energy inputted into the system should exceed the cavitating threshold wattage (CTW) throughout the working volume (m^3^) of the liquid. As reported in literature, the CTW range can be relatively broad which is conditional upon liquid characteristics, in particular its viscosity (15–65 kW·m^–3^).^171^ Yet, as with many turbulent regime tanks, viscous effects are expected to be negligible at high Reynolds numbers (Re ∼ 10^4^ - 2 × 10^4^).^172,173^

UAMs have become increasingly common in sonochemistry and synthesis/extraction processes. Ultrasonic powers in the 0.02–0.1 MHz range have proven effective for coating passivation and facilitating chemical reactions. Interestingly, Kumar and Maurya^174^ have documented a high yield method for the synthesis of Hantzsch esters and polyhydroquinoline derivatives within aqueous micelles via sonochemical irradiation. Moreover, high intensity UAMs have favored the fabrication of novel nanomaterials, such as amorphous metal nanoparticles from organometallic precursors (i.e., Fe(CO)_5_ or Cr(CO)_6_) and molecular crystals in a time effective manner without high pressures or temperatures.^175^ Similarly, UAMs have been employed in food, drug delivery and allied industries, as well as for the extraction of bioactive substances at different frequencies.^176^ Ultrasounds can opportunistically penetrate the matrix of various biological/synthetic components to trigger the release/extraction of the value-added substances contained within. Xu and co-workers^175^ have provided a critical review on UAMs in the food industry, which also highlights relevant process conditions. Specifically, both batch and continuously stirred configurations have been used in the 20–2400 kHz frequency range for the extraction of almond oils (batch 20 kHz), polyphenols and caffeine (40 kHz), and herbal oils (0.4–2.4 MHz).

**Figure 9:**
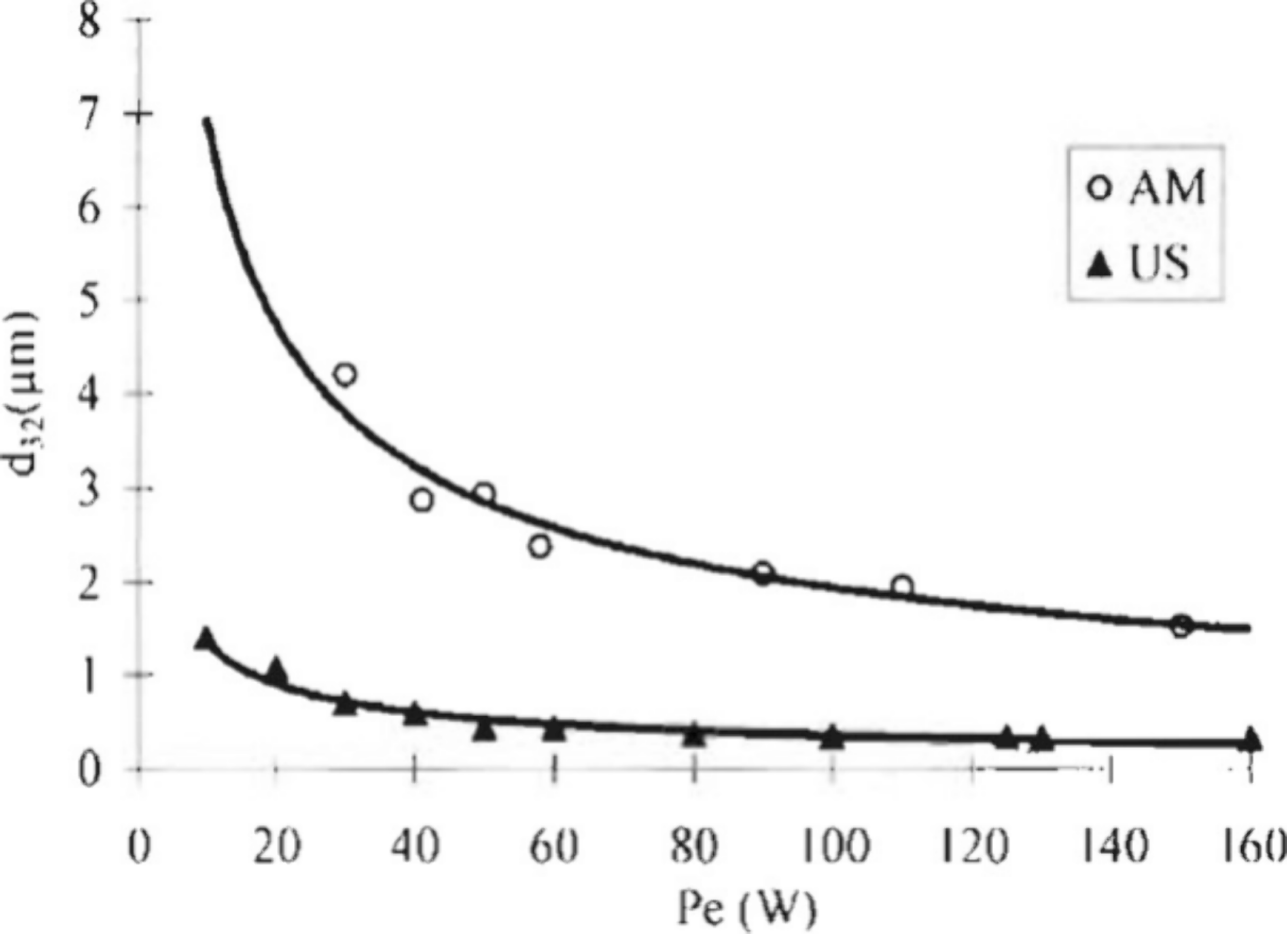
Effect of the inputted power (Pe) on the Sauter diameter (*d*_3,2_) of droplets using mechanical agitation (MA) and ultrasound-assisted (US) methodologies. Reprinted with permission from ref (178). Copyright 2002 Taylor and Francis Publishing.

UAMs have been employed to generate emulsions by propagating ultrasonic waves (>20 kHz) within liquid–liquid biphasic systems.^177^ Acoustically induced cavitation and the corresponding power dissipation are the leading factors in the breakup mechanism of a dispersed liquid phase into droplets.^178^ Calligaris et al.^177^ provided an overview of ultrasonically assisted emulsification processes in comparison with high pressure homogenization (HPH), and their combination. Interestingly, it was determined that a stable nanoemulsion of sunflower oil in water (*d*_3,2_ ∼ 151 nm) required 360 MJ·m^–3^ via HPH, which was around 5-fold greater energy than that needed to obtain a similar emulsion using UAMs (75 MJ·m^–3^). Additionally, the presented data suggested that the combination of UAMs and HPH was beneficial to further reduce the energy demand to 48 MJ·m^–3^, while achieving even smaller droplets (121 ± 4 nm). As reported in literature, relatively extended insonation intervals (time that materials are subjected to ultrasound for) and broader amplitude ranges may lead to finer emulsions as a greater energy is inputted into the system. Yet, there is an optimum power so as to avoid coalescence. Canselier et al.^178^ compared the effect of energy input on the Sauter diameter of droplets using mechanical agitation and UAMs (Figure 9). It is noteworthy that the emulsified systems were obtained at a lab scale, thus scaling-up may not be straightforward. It is well understood that even with multiple ultrasonic cuphorns located equidistantly in a large vessel, an inhomogeneity of the acoustic field is likely to occur due to the rapid absorption and subsequent attenuation of the ultrasonic wave within the liquid medium.^178^

Other than emulsification and extraction, novel UAMs have been proposed for the encapsulation of volatile or poorly stable ingredients, with specific emphasis on functional foods and bioactive compounds. This was fulfilled by combining UAMs with spray-drying. Interestingly, liquid cheese aroma was encapsulated within a maltodextrin matrix via two different emulsifying methods, namely UAM and Ultra-Turrax treatments, both followed by spray-drying^179^ When comparing the encapsulation efficiency of the ensuing microcapsules, it was found that the microcapsules undergoing UAM treatment (at a frequency of 20 kHz with a maximal power output of 600 W operated over 5–20 min) were capable of retaining up to ∼20% more cheese aroma.

In 2018, Ruiz-Montañez and co-workers^180^ employed an UAM to encapsulate miglyol-dissolved jackfruit bioextracts within a shell made of maltodextrin, with antioxidant and antiproliferative activity. In 2021, core–shell microcapsules laden with sun-blocking metabolites (octyl methoxycinnamate) within oligomeric proanthocyanidins were achieved by an ultrasound triggered LbL microencapsulation procedure at an ultrasonic power of 0.4 kW over 5 min.^181^ Similarly, Cimino et al.^182^ prepared core–shell microcapsules with a blend of soybean oil and calciferol as the active and naturally sourced glycogen nanoparticles as the shell, using a rapid (45 s) dip-in probe UAM with power of 0.16 kW. In 2022, Li and co-workers^183^ reported the encapsulation of natural nutritional pigments, betalains, with maltodextrin by UAM (0.2–0.4 kW over 5 min) leading to an encapsulation efficiency of ∼80%.

### 3.3 Membrane Emulsification

**Figure 10:**
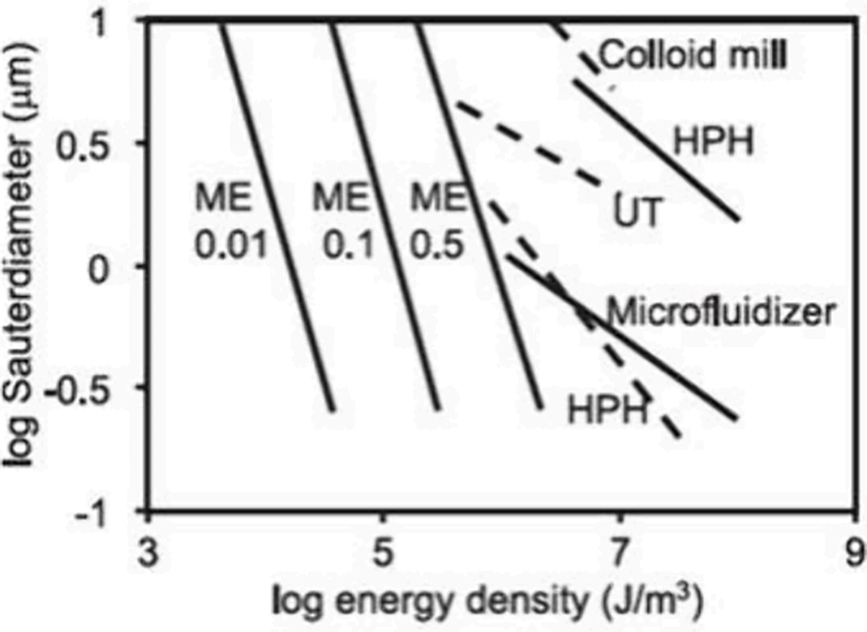
Energy usage of various emulsification techniques. HPH is high-pressure homogenization, UT is ultraturrax, ME is membrane emulsification (cross-flow) with the associated numbers representing dispersed phase fraction. Reprinted with permission from ref (186). Copyright 2004 Elsevier.

ME is an advancing method of emulsification, first reported by Nakashima and Shimizu around 35 years ago.^184,185^ This technique allows the formation of well controlled emulsions with relatively little energetic input when compared to traditional homogenization techniques (Figure 10). At the time the scalability of this technique was questioned due to the requirement of well controlled membranes and flow rates. However, engineering solutions have resulted in improvements in these facets, allowing for scalability to be achieved, as outlined below. In this process the dispersed phase is pumped through a porous membrane of known and controlled pore size into the continuous phase containing an emulsifier which is either being sheared by an impeller or allowed to flow across the membrane surface. These are referred to as stirred membrane and cross-flow ME, respectively.

**Figure 11:**
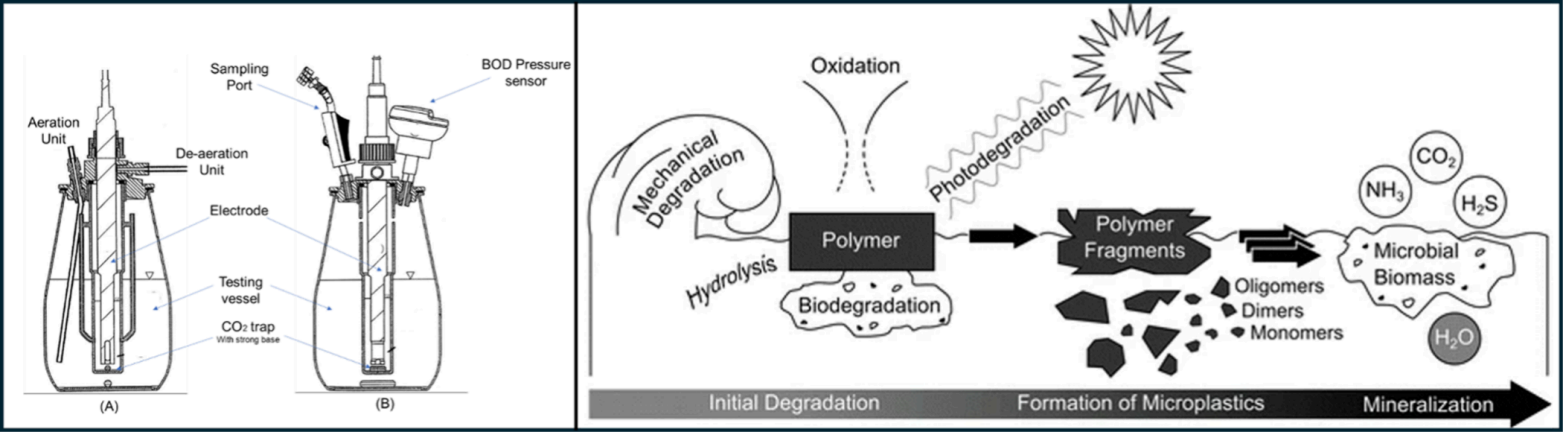
Typical testing apparatus for (left, A) the online CO_2_ evolution test and (B) multicomponent biodegradation test system (BOD, DOC, and CO_2_). Reproduced with permission from ref (224). Copyright 2004 American Society for Microbiology. (Right) General polymer biodegradation scheme in aquatic environments. Reproduced with permission from ref (225) under Creative Commons License 4.0 (http://creativecommons.org/licenses/by/4.0).

As an example, a standard gear pump operating at 1.6 A and 10 V will result in an energy usage of 16 J·s^−1^ for a modern membrane device such as the Micropore AXF-1,^187^ which requires two of these pumps to reach the maximum stated process rate of 200 L/h of emulsion. This equates to 5.8 × 10^5^ J·m^−3^ of emulsion produced. However, as described in Figure 11 the targeted diameter plays a significant role in the energy usage due to the impact of the required flow/shear rates and the membrane pore size. In the case of cross-flow emulsification, slower flow rates generally lead to larger emulsion droplets as the droplets are detached from the membrane by the shear stress provided by the continuous phase flow.^188^ This increase in droplet size continues until a plateau is reached.^189,190^ It is important to note that defects in the membrane pores, targeted dispersed phase volume and the pore size/distribution may also affect the required minimum flow rate to produce emulsions of a narrow size distribution.^186,188,191^

These effects are not restricted to the cross-flow process. Indeed, in stirred cell ME the shear stress applied to detach the droplets, which depends on the impeller speed, also plays a crucial role. A complex empirical relationship is proposed by Kosvinstev et al. that relates the resulting droplet size with the water/oil surface tension, membrane pore size and applied shear rate.^192,193^ In this case the shear stress is primarily due to the impeller as it provides both the initial mixing force and mechanism of detachment of the forming droplets from the membrane.^192^ For a given pore size, impeller blade geometry and oil/water system, the droplet diameter decreases exponentially as the shear rate or energy input is increased.

Given that stirred cell emulsification throughput is limited by the volume of the cell, the number of complete processes that are required to reach a desired volume is equipment dependent. In the case of a laboratory scale device with a dispersed phase flow rate of 0.5 mL/min and a cell volume of 0.1 L, the energy required to form droplets of 80 μm diameter using a standard system consisting of an alkane being dispersed in water (*γ*_*ow*_ = 52 mN/m), a membrane of pore size 10 μm, and impeller of critical radius of 11.5 mm, is 6.5 × 10^7^ J·m^–3^.

Both ME methods require significantly less energy than traditional emulsification techniques, such as ultrasound and mechanical homogenization (Figure 9 and 10). Despite this, the use of ME is not common in industry, since the conditions that work well on a small laboratory scale may not be easily translated to larger industrial processes. Furthermore, due to the number of factors affecting the emulsion size and size distribution, as well as the potential for pores becoming clogged, ME is arguably more complex. However, the use of and research into ME is increasing^191,194^ since it offers clear advantages of lower energy requirements, and increased ability to use sensitive ingredients that may spoil when subjected to classical emulsification techniques–factors especially important within the food industry.^194,195^ Examples include work by Katoh et al., who were able to produce a spread consisting of 25% fat,^195^ as well as work by Piacentini and co-workers who demonstrated the use of ME to produce microcapsules of biophenols from olive mill wastewaters.^196^ In addition to this, recent work using cross-flow ME has focused on the production of capsules for self-sealing cement.^197,198^

It should be noted that the energetic considerations discussed here are not exhaustive, indeed, we only highlight the emulsification techniques to prepare a microcapsule product template. Additional energetic costs associated with post emulsification treatment for shell synthesis have not been included in this analysis, as they are independent of the method of emulsification.

## 4 Regulations and Biodegradation

Microcapsule walls are traditionally poorly degradable plastics, which can pervasively accumulate once released into the environment.^199^ Furthermore, many materials within the academic literature are claimed to have biodegradability or be initially composed of biodegradeable constituents. However, when these materials are formulated into capsules, their biodegradability is frequently not assayed, which is an integral piece of missing information as degradation characteristics may significantly change during the encapsulation process. In light of the above, it is pivotal to differentiate between the natural degradation of (bio)polymers and the degradability of the corresponding microcapsules. According to European regulations, the biodegradability of a polymer does not inherently imply that capsules with shells made from the same polymer would exhibit similar biodegradability, especially if they have been chemically modified in the process (e.g., through cross-linking as outlined above).^36,200,201^

In general, spent microcapsules as well as cosmetic microbeads commonly found in toothpastes, cleansing body lotions, and skin exfoliating formulations, are a common source of anthropogenic MP debris.^202,203^ MPs have been reported ubiquitously within the marine ecosystem, thereby becoming bioavailable to many species, which has posed a severe risk against the conservation of wildlife and complex ecological systems.^204^

Albeit indirectly, the United Nations Sustainable Development Goals are proactively engaged in the global challenge of MP pollution. Goal 6, ‘Clean Water and Sanitation’ squarely addresses the containment of hazardous chemicals and MP pollution in aquatic environments (Target 6.3). Additionally, Goal 12 calls for a ‘Responsible Consumption and Production’ to curtail plastic waste generation while advocating for circular recycling practices (Target 12.5). This approach is pivotal in averting the generation and unbridled dissemination of plastic debris into marine habitats (Goal 14, “Life Below Water”), which ultimately leads to MP formation (Target 14.1). Furthermore, the sustainable management and preservation of terrestrial and freshwater ecosystems is imperative (Goal 15, “Life on Land”), which are often affected by MP pollution (Target 15.1).^37^

Secondary MPs typically originate from the embrittlement of larger synthetic litter (e.g., textiles, tires, single-use polyethylene (PE) bags) into smaller (≤100 μm) fragments due to the synergistic action of factors, including chemical bond breaking, photocatalytic and biochemical activities as well as mechanical erosion.^205,206^ Moreover, harmful pollutants (e.g., aromatic hydrocarbons, heavy metals, manmade polychlorinated biphenyls, polybrominated diphenyl ethers, endocrine-disrupting poly fluoroalkyls) can become adsorbed to the surface of MPs which may pose a threat for bioaccumulation and biomagnification in aquatic-to-human food chains (i.e., trophic transfer from wild-caught fish to human beings).^207,208^ To date, human exposure to MPs has been documented with MPs being detected in human excrement and organs, such as placenta.^209^ In light of the above, global concerns over the environmental, health and safety implications of the unbridled breakout of MP pollution have recently arisen.^210^ It has been reported that primary MPs account for <10% of the overall yearly nonrecoverable plastic pollution; however, their absolute estimate is ∼7.5 tonnes/year exclusively from personal care products and up to 176,000 tonnes in total within the European Union (EU). This MP pollution represents a severe ongoing environmental concern that is not yet fully regulated.^206,211^ Given the pace at which the use of MPs is spreading worldwide, immediate global action is required. Below we provide a framework of the upcoming regulatory legislation toward addressing the production of MPs, as well as an in-depth overview on the current challenges around developing novel biodegradable microcapsules.

### 4.1 Cosmetics

Recently, the beauty industry has come under increased scrutiny due to its large variety of cosmetic products being enriched with MP-based microparticles.^36,200,203^ As documented by the European Chemicals Agency (ECHA), many beauty and personal care products are a primary source of MPs since microscaled polyethylene terephthalate (PET) particles/capsules are intentionally added for both functional and aesthetic purposes. Specifically, lip balms, lotions and creams are classed as leave-on cosmetics which likely end up as landfill waste via household waste streams, once wiped off by the user via mechanical action.^203^ In contrast, exfoliating and cleansing formulations are typically rinse-off cosmetics which are discharged down the drain after a single use, thereby entering the wastewater system.^201^ Although a few advanced wastewater treatments have proven effective at recovering up to 90–95% of suspended PE, polypropylene (PP), and PET fragments with an average size of ≥100 μm from urban influents/effluents, they can be complex and costly to operate.^212^ In addition, at present the majority of sewerage treatment plants do not appear to integrate such technologies. This would require substantial infrastructural enhancements, possibly spanning over several decades to implement, as well as requiring unprecedented allocation of funding by global councils.

Moreover, it is unclear whether MPs smaller than 100 μm, as well as their fragments (nanoplastics), can be recovered as efficiently as larger MPs. These smaller particles represent the largest portion of industrial MPs, especially from cosmetic, textile and laundry industries.^13^ Laundry detergents, fabric softeners,^13,61,122^ rinse-off and leave-on cosmetics formulations may include MPs as encapsulants (shells) for controlled release, with commercial sizes typically around 20–50 μm.^108,199,201^ These have been detected in wastewater, and, also, in fresh and marine environments.^205^ In recent years, global media, academic, intergovernmental and nongovernmental organisations have attempted to raise awareness within the sector to phase out added MPs and have urged legislative bodies to act immediately and proactively.^213^ Current work within this sphere is focused on assessment of the environmental and human health risks associated with unregulated MPs discharge, as their biodegradation is currently unfeasible, and cost-effective recovery technology is hitherto unavailable.^199^ Although the EU Commission responded promptly by enacting a partial restriction against cosmetic rinse-off microbeads in December 2014 (2014/893/EU), no enforceable EU-wide ban arose.^214^ Specifically, ecological criteria for the award of the “EU Ecolabel” were established toward promoting environmentally friendly products over those with a suspected high environmental impact (e.g., MPs-enriched products). Recently, increased public and governmental awareness have motivated the EU Commission to repeal 2014/893/EU in favor of a more comprehensive directive (2021/1870/EU). This came into effect in October 2021 to cover human and animal care products (i.e., leave-on and rinse-off formulations) for both private and professional use.^215,216^ In addition, multiple EU beauty industries claimed to have voluntarily reduced the use of microbeads in their products by up to ∼95% between 2012 and 2017 indicating that the use MP beads were gradually being decreased due to the self-regulation endeavors from the international cosmetic market.^36,217^

### 4.2 International Regulations

Outside of the EU, the UK is acknowledged to have enacted the most far-reaching ban in 2018 against water-resistant and synthetic microparticles in the industrial market.^203,218^ Motivated by the EU-UK approach, many other countries, such as Canada (enacted 2019) and New Zealand (enacted 2018), have adopted dedicated measures, and have applied similar bans to tackle the rapid spread of MPs into the environment.^203^ Notwithstanding, there has appeared to be a lack of general agreement on the definition of MPs, resulting in other countries such as China and India acting independently, thereby imposing self-standing tolerance limits, which can differ significantly from other nation’s legal regulations.^219,220^ This international concern requires a multistakeholder approach and harmonization of the existing policy instruments in order to mitigate the proliferation of MPs worldwide, since the long-term implications of MP pollution should be taken into account.^221^

To this end, ECHA has proposed a comprehensive ban under the Registration, Evaluation, Authorisation and Restriction of Chemicals (REACH) regulation [EC 1907/2006]. If implemented, this may represent the strictest regulation to curb the uncontrolled emission of primary MPs within the industrial, professional, and end-use consumer spaces so far. In response to the increasing calls for effective regulatory directives against the free spread of MPs in the environment, an up-to-date annex defining the new rules in the field of *synthetic polymer microparticles* was, in September 2022,^222^ presented in a discussion paper by the EU Commission This document encompasses the key issues associated with MPs at an industrial, circular economic, socio-environmental, and political level. It has been stated that more than 42,000 tonnes of MPs end up in the environment annually within the EU. Concerning a ban on microbead enriched products (i.e., rinse-off cosmetic products or detergents), no official transition period has materialized since industries were ready to eliminate MPs based particles/capsules formulations by 2020. When specifically dealing with synthetic polymer microparticles (i.e., shells) encapsulating fragrances, a five-to-eight-year transition period has been considered for industries to mitigate the potential risks of reduced revenue. However, this document is still pending and being assessed by EU member states.

### 4.3 Biodegradation Standards

The same document also establishes the key rules to prove biodegradability of synthetic polymers as the precursors of microcapsule shells. Specific biodegradability methodologies were designed to quantify biotic degradation while being cognisant that abiotic degradation may also occur. In recent decades, both industrial and scientific communities have developed complex standardized testing hierarchies to assess the biodegradability of specific chemical substances, with particular emphasis on the Organisation for Economic Co-operation and Development (OECD), the International Organization for Standardization (ISO), and Comite Europeen de Normalization, (CEN). Most importantly, the OECD system has been rigorously implemented by many EU countries. In their recent review, Strotmann and co-workers have focused on the technical strengths and limitations of current biodegradability testing methods.^223^ The typical biodegradability testing setup for online CO_2_ evolution and multicomponent biodegradation (CO_2_, biochemical oxygen demand (BOD), dissolved organic carbon (DOC) are displayed in Figure 11.

Approval is awarded to those polymers capable of meeting biodegradability criteria in specific environmental compartments, such as fresh, estuarine, marine waters, marine water/sediment interface, and soil. Based on their underlying rationale and applicability assessed by the OECD, specific tests are intended to prove the biodegradability of polymers via the respirometric evaluation of CO_2_ (60% mineralization) produced aerobically over a minimum of 28 days up to 60 days in a liquid environment (OECD 301B) and within sealed vessels (headspace test OECD 310). Biodegradation tests are also run in marine environments (OECD 306) to investigate the biodegradation process of a material in seawaters.^226^ Furthermore, inherent biodegradability tests are conducted (MITI Test II OECD 302C) for determining the biodegradability of formulations typically not readily biodegradable, with a stricter pass criterium to achieve ≥70% mineralization (consumed O_2_ or evolved CO_2_) within 14 days.^227^ When considering buoyant synthetic materials, similar aerobically driven biodegradability tests have been adopted, such as the evolved carbon dioxide (ECD) assay (EN ISO 14852:2018) and the chemical (COD) and biochemical oxygen demand (BOD) analysis in a closed respirometer (EN ISO 14851:2004).^222,226^ Slight guideline modifications may occur depending on the receptor environment in which nonfloating plastics are assayed, such as seawater-sediment interface (EN ISO 19679:2016), soil (EN ISO 17556:2019), marine sediments (ISO 22404:2019) via ECD, and seawater-sandy sediment interface via COD-BOD (EN ISO 18830:2016). Ultimately, the degradation associable with plastic based materials is ≥90% (pass criterium) within 6 months in aquatic environments and/or 24 months in soil, sediment, or water/sediment interface tests. However, a relatively extended degradation time window is allowed for polymers used in products for agri-horticultural applications, namely 12–16 months and within 48 months (after the end of product functionality period) in water and soil compartments, respectively. Synthetic polymers for (micro)encapsulation should be assayed for their degradability in the form placed on the market (the organic core may be replaced by an inert material such as glass particles) or as an isolated coating (shell-only hollow microcapsules). Such degradation rates are reasonably limited when compared to that of recalcitrant hydrophobic materials, such as low (LDPE) and high-density polyethylene (HDPE), typically used for plastic bags and beverage/laundry containers, with an estimated half-life of more than 250 years at landfill-soil compost conditions.^228^ In addition, LDPE sheets underwent only marginal biodegradation (partial whitening, possibly due to the intermetabolic activity of filamentous fungi) with negligible weight loss when buried in moist soil compartments continuously for over 32 years.^229^ In contrast, no evidence of biodegradative activity was found for polystyrene, polyvinyl chloride, and formaldehyde cross-linked urea resins.^230^ Indeed, the latter possess chemical structures relatively similar to melamine formaldehyde resins, which are extensively used in industry to fabricate microcapsules laden with perfume for laundry formulations,^13^ and insecticides for agri-horticultural purposes.^110^ These aforementioned microcapsules with synthetic shells accumulate pervasively into the environment once released, with the former (laundry) entering the aquatic environment irreversibly, whereas the latter (agri-horticultural) persisting within the soil.^199^ For agri-horticultural use, microcapsules may require a high degree of aldehyde driven cross-linking, which is critical to preserve their stability when exposed to adverse climate conditions (UV radiation, erosion, etc.). For laundry applications, the capsules may be subjected to high temperature and shear during washing-drying cycles, hence a significant degree of shell cross-linking is required to prevent any core leakage from perfume microcapsules during the wash cycle.^13^

### 4.4 Response from Industry

In response to the ECHA restrictions that were ratified in 2022, several companies have already taken proactive measures to offer innovative and scalable solutions ahead of the forthcoming ban on nonbiodegradable MPs due for enforcement by 2027. In December 2021, Givaudan (Zurich, Switzerland) unveiled PlanetCaps which are the first-ever biodegradable fragrance capsules designed for fabric conditioners.^231^ PlanetCaps entirely rely on bioderived ingredients in compliance with ISO16128, with more than 50% renewable carbon. Interestingly, Givaudan asserts that the biodegradability of their new product is certified by OECD testing criteria, yielding over 60% of the shell decomposed within 60 days of their usage. In May 2022, Iberchem (Spain, EU) launched an innovative biodegradable fragrance capsule technology, namely VernovaCaps, formulated with a minimum of 60% biobased materials, with potential for fabric softeners and personal care.^232^ VernovaCaps shells have been reported to meet the rigorous OECD standards of more than 60% ‘*Readily biodegradable’* within 28 days *in toto*, with additional degradation achievable beyond this time frame. Later in November 2022, Firmenich (Zurich, Switzerland) launched PopScent EcoMax capsules, which are composed entirely of biodegradable ingredients and hold significant potential for laundry applications.^233^ Similarly, MikroCaps (Ljubljana, Slovenia, EU) developed a new microencapsulation system, known as Biocaps, to encapsulate fragrances. These Biocaps were proven highly biodegradable via OECD testing (shell decomposition greater than 60% within 28 days). Additionally, they exhibited excellent resistance to high temperatures and pressures, making them suitable for a plethora of applications, such as fabric softeners and cosmetics that require specific release mechanisms (e.g., burst release or long-term release by diffusion).^234^ As of April 2023, MANE (Gladstone, Michigan, US) introduced an innovative line of eco-friendly fragrance microcapsules i.e. Manecaps for fabric softeners. These have demonstrated more than 60% biodegradability within 28 days without impairing the hedonic sensory perception of freshness once the fragrance has released.^235^ Although significant advances have been reported by various microencapsulation companies toward meeting the current and future demands for greener products, the available information on the release, sensory, mechanical, and adhesive properties of these newly developed microencapsulating systems remains limited. Consequently, there appears to be no direct pathway to assess the overall performance of these new microcapsules and their efficacy in comparison with the well-established synthetic microcapsules.

### 4.5 Potential Alternatives

Other than melamine-formaldehyde and those outlined in Section 4.4, alternative polymers as microcapsule shells have been investigated for industrial-like applications over the years, including polysulfone,^236^ polyurethanes-urea, polyacrylamide, poly(methyl methacrylate) and functionalized polyesters.^62,237^ These materials were proven to provide desirable performance properties, such as thermostability, mechanical toughness and sustained release.^238^ However, these shells are not inherently biodegradable, which hinders their potential implementation/applications.^113,239^ New bioinspired semisynthetic polymers, containing polylactic acid (PLA), PLGA, polycaprolactone and aliphatic polycarbonates, have emerged as biobased shell-building blocks for a number of uses, including biomedical applications such as drug delivery, imaging and antioxidant delivery, due to their biocompatibility, and biodegradability.^240−242^ Other semisynthetic polymeric wall ingredients are cellulose derived.^46^ Among all the available derivatives, ethyl cellulose has garnered great interest as a hypo-allergenic and eco-friendly drug encapsulation vehicle.^243,244^ Similarly, nanocrystalline cellulose has been used to entrap model fragrances, such as β-damascone.^245^ Indeed, carboxymethylcellulose-based hydrogels have been reported for the controlled delivery of active molecules, including rhodamine B dye, isoliquiritigenin, or lysosomes.^246^ Although promising, no large-scale encapsulation technology has been developed so far, possibly due to the sensitivity of cellulose-based microcapsules to temperature, acids, bases, and organic solvents.^46^

More specifically, cellulose-based microcapsules can be sensitive to relatively high temperatures, especially if only level cross-linking has been performed. Excessive heat, as well as strong acidic environments, can also lead to degradation of the cellulose structure. In contrast, it is relatively stable in basic conditions although strong concentrated bases can still lead to the degradation of its structure over time. As with many other pH-responsive polysaccharides, cellulose can either accept/donate protons in response to the fluctuation of the environmental pH, resulting in conformational, structural chain, and surface activity changes.^246^ Due to its high polarity, cellulose is generally insoluble in most organic solvents, with the exception of chemically complex solvents like dimethyl sulfoxide and *N*-methylmorpholine-N-oxide.^247^ Additional natural macromolecules have been reported for fragrance encapsulation, with particular emphasis on chitosan, cyclodextrin, and shellac. Specifically, both crustacean and fungal chitosan types have been used for the encapsulation of hydrophobic Miglyol 812N (dermal and oral applications) and hexyl salicylate, respectively.^122,124^ Shellac combined with whey protein isolate has also been employed for the encapsulation of probiotics.^248^ Additionally, cyclodextrins have emerged as valuable fragrance carriers owing to their core-hosting performance and biocompatibility, as reported by Azizi et al.^249^ having fabricated β-cyclodextrin based microcapsules through the polycondensation of 4,4′-methylene-bis(phenyl isocyanate) with β-cyclodextrin at the oil/water interface for the sustained release of floral scent nerolin. Such microcapsules relied on biosourced cyclodextrins instead of the traditional diols often utilized in interfacial polycondensation. These microcapsules, with a unimodal volume-based particle size distribution (average size ∼11 μm), were impregnated onto polyamide knitted fabrics (substrate) within a bath containing an acrylic cross-linker, and subsequently thermally fixed at 120 °C. The resulting impregnation yield was as high as ∼74%, as confirmed by scanning electron microscopy (SEM). Interestingly, the microcapsules exhibited high adhesiveness to the substrate even after 35 washing mimicked cycles, making them of interest to the cosmetics and textile industries. More specifically, microcapsule adhesiveness refers to the ability of microcapsules to adhere onto a target surface/substrate. Therefore, controlling the adhesiveness of microcapsules is also paramount in certain applications where controlled release onto a specific substrate is required. In such cases, stimuli-responsive shell materials can be engineered to achieve the desired release behavior to maximize the functionality and performance of the microcapsules.^199^

When considering biodegradability and environmental preservation, the utilization of natural inorganic materials (Table 3) for the microcapsule shell is drawing interest owing to their excellent physicochemical properties and broad-spectrum applicability.^46^ Several inorganic materials with a potential for encapsulation have been reported, such as phosphates, clays (aluminum phyllosilicates), calcium carbonates, and silicates.^250,251^ Indeed, microcapsules with mineralized SiO_2_ shells are being intensively investigated due to their cost-effectiveness and environmental harmlessness. A sol–gel methodology to grow SiO_2_ shells around active-loaded cores has long been established.^252^ Single, multiple, and Pickering emulsion technologies followed by the precipitation/accretion of SiO_2_ nanoparticles on the core surface (often an oil–water emulsion) have proven successful.^253,254^ Alternatively, Xue and co-workers^255^ have developed silica nanocapsules laden with lemon essential oil using hollow mesoporous silica nanoparticulates (payload ∼86%) for superhydrophobic aromatic cotton fabrics. Remarkably, the oil loss in static conditions after 6 days was below 10%. More recently, Yeom et al.^60^ have fabricated silica microcapsules with a core–shell structure for prolonged fragrance retention by o/w emulsion template synthesis using oil-soluble tetraethyl orthosilicate (TEOS). Seeded growth of silica crystals around hexyl cinnamaldehyde cores was achieved leading to shell thicknesses between 42 and 70 nm, which is similar to those reported for synthetic melamine-formaldehyde microcapsules.^138^ Interestingly, the encapsulated fragrance was retained for ∼80 days despite the microcapsules being exposed to harsh laundry-like conditions of 15 wt % sodium dodecyl sulfate at 60 °C. The authors also claim that these capsules are environmentally friendly, however no demonstration of their degradation is reported. Other materials have also generated research interests for microencapsulation including calcium carbonate and phosphate which are inexpensive, ecologically benign, and pH responsive.^256^ As reported in literature, Wang and co-workers^257^ have fabricated food-grade core–shell CaCO_3_ capsules via Pickering emulsion templates to encapsulate limonene. These capsules exhibited release profiles that could be controlled using pH (faster release due to capsule degradation at low pH) on relatively short time scales (less than 1 h). These capsules are proposed for use as encapsulants in the food industry that could protect their payload until reaching the acidic conditions found in the stomach.

**Table 3:** Summary of Biodegradable and Biomimetic Microcapsules with a Potential for Industrial Applications

core	shell	size (μm)	proposed application(s)	year	reference
Hexyl cinnamaldehyde	Silica	1–2	Cosmetics/Laundry	2022	(60)
Hexyl salicylate	Fungal chitosan–gum Arabic	∼35	Detergents/Cosmetics	2021	(122)
Miglyol 812N	Crustacean chitosan–gum Arabic	5–10	Dermal and oral care	2014	(124)
Curcumin	Ethyl Cellulose	∼50	Pharmaceutical	2022	(243)
Linseed oil	Ethyl Cellulose	0.03–400	Pharmaceutical	2018	(244)
Probiotics	Shellac-protein isolate	10–20	Nutraceutical	2022	(248)
Nerolin	Cyclodextrin	∼11	Textile	2019	(249)
Lemon essential oil	Silica	<1	Textile	2016	(255)
Fragrance	Calcium carbonate	20–40	Food/Pharmaceutical	2012	(257)
Insulin	PDA	<20	Biomedical	2015	(260)
λ-cyhalothrin	PDA	<1	Pesticidal	2018	(262)
Dextran, 5,6-carboxyfluoresceine and dexamethasone	PSS, PAH and PLGA “lid”	1–11	Pharmaceutical	2022	(274)
Saflufenacil	Silk fibroin	3	Pesticidal	2022	(275)

To date, substantial premature loss of the encapsulated active from CaCO_3_-only shells has been observed^258^ possibly due to the porous nature of CaCO_3_. Therefore, polymeric-inorganic composite systems have also been explored to provide microcapsule shells with advantages from both materials. In 2009, Long et al.^256^ seemed to have pioneered this field reporting successful shell composite microcapsules composed of melamine-formaldehyde (organic coating) mechanically reinforced by deposition of CaCO_3_ crystals (inorganic coating). Although they demonstrated excellent barrier properties, the microcapsules were partially fabricated with a synthetic polymer (melamine-formaldehyde), hence no degradability can be achieved naturally.

A monomer that is growing in research interest is dopamine and the polymeric PDA has been used for microencapsulation.^259^ Bioinspired by the extremely adhesive properties of proteins (i.e., catechol-amine moieties) in mussels, dopamine is a promising biomimetic catechol-amine rich monomer, which may form homogeneous layers onto many surfaces. Accordingly, Wang and co-workers^259^ have produced bioadhesive microporous PDA architectures onto polystyrene microcapsules and titanium substrates via LbL self-assembling of PDA, with potential for biomedical applications. Kang et al.^260^ have fabricated PDA microcapsules for insulin delivery via a facile and inexpensive route for chemical oxidative self-polymerization of dopamine onto specific sacrificial templates of manganese carbonate microparticles. Dopamine was also proven to polymerize onto emulsions stabilized with cinnamoyl chloride modified cellulose nanocrystals with a potential for essential oil and pesticide encapsulation.^261^ Similarly, Zou et al.^262^ have produced PDA-coated microcapsules by self-polymerization of monomeric dopamine as a vehicle for the sustained release of hydrophobic pesticides, such as λ-cyhalothrin. In addition, microcapsules with an ultrathin PDA shell (∼48 nm) and a hierarchical structure were developed for the immobilization of multienzymes utilizing a metal organic framework as the template.^263^ Overall, PDA has proven relatively effective at forming outermost microcapsule coatings, hence reducing the leakage of active ingredients. PDA also demonstrates substantial adhesive properties and may adhere firmly to porous, wrinkled, and smooth surfaces. Although claims for lab-produced PDA as a fully biodegradable material are widespread in literature, very little is known on its possible degradability and mechanism thereof, which remains to be investigated, and should be tested using the internationally recognized methods outlined above.^264,265^ An additional industrial consideration is the scalability of any proposed environmentally friendly capsules. Some of the capsules described throughout the literature utilize LbL,^259,266,267^ multiple emulsions^143^ lithographic or 2D templates.^268^ However, LbL and lithographic techniques suffer from similar limitations regarding scale. Current LbL techniques require time-consuming consecutive immersion steps in alternating charged species (including polyelectrolytes) and thus low efficiency and yield.^269^ Similarly template-based microencapsulation techniques such as soft lithography often require bespoke templates. This allows for substantial control and reproducibility of formulated capsules, but at a relatively high cost. Indeed, to our knowledge, industrial scale up of this technique for encapsulation has yet to be achieved.^270,271^ Multiple emulsions are also potentially promising, but due to their inherent complexity are difficult to implement on scale. For example, in the case of a water/oil/water emulsion, destabilization and coalescence may occur in the innermost water phase. Alternatively, these inner droplets may escape into the continuous phase, and release may be driven by osmotic pressure.^272^ Furthermore, the number of droplets present within each larger droplet may influence the release rates and the stability of the resulting capsules.^273^ Thus, load distribution and delivery efficiency may become compromised when attempting to implement these multiple emulsion systems in a commercial setting. Therefore, these techniques have, to our knowledge, not been widely implemented industrially however this may need to be revisited to adapt to incoming legislative requirements and energetic considerations.

## 5 Promising Microcapsules: A Feat of Microengineering

The microcapsule industry is undergoing a transformative period. Next generation microcapsules will have to be produced using low-energy manufacturing solutions and biodegradable materials while maintaining their impermeable characteristics, sufficient mechanical strength and potential for targeted release. In this section, we outline several promising recent works that demonstrate some of these qualities in order to highlight potential pathways for future research. It should be noted that many authors report biodegradability qualitatively (if at all) and as such assessing their biodegradability would be a necessary requirement to implement these methods further.

**Figure 12:**
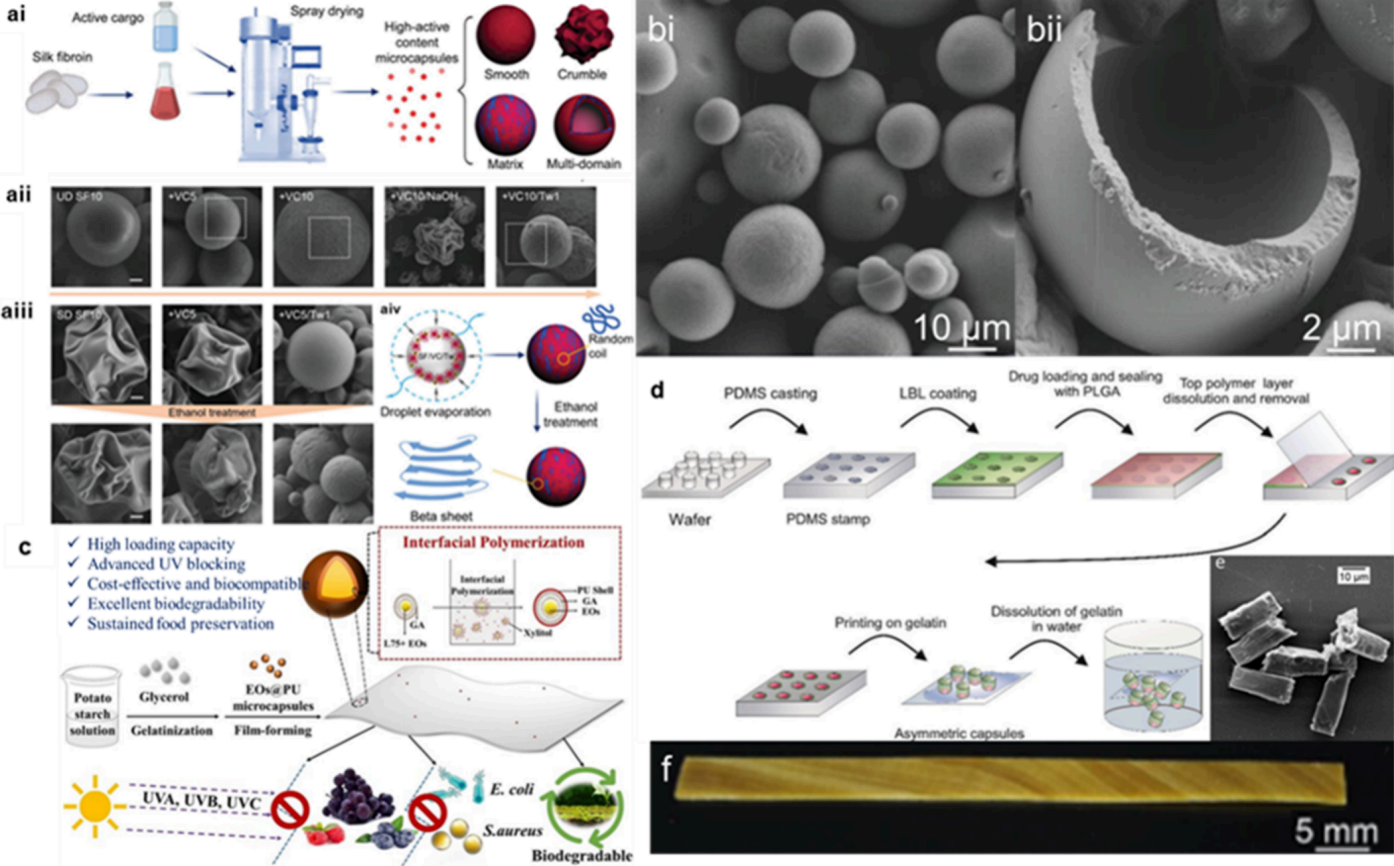
Promising microcapsules discussed within this review. (a) Structural and configurational manipulation of suspended silk fibroin into microcapsules with different morphology: (ai) Schematic of the lab-scale production of different types of microcapsules, including smooth/crumpled surfaces, matrix, and multidomain configurations. The red spheres represent the silk fibroin matrix, whereas the blue streaks indicate the active cargoes. (aii) Morphology and surface topography of ultrasonically freeze-dried microcapsules (scale bar, 10 μm). (aiii) Morphology and surface topography of spray-dried microcapsules (e; scale bar, 2 μm) before [(aiii) upper panel] and after hydroalcoholic treatment [(aiii) lower panel]. (aiv) Schematic of a silk fibroin microdroplet into microcapsule. (b) SEM micrographs of microcapsule microcapsules with a silk fibroin shell; (bii) cross section of an incomplete spray-dried microcapsule with a homogeneous silk fibroin shell; adapted from with permission from ref (275). Copyright 2022 Wiley-VCH. (c) Biodegradable, biocompatible, and cost-effective food packaging films containing Eos@PU microcapsules with sustained slow-release and excellent UV-blocking properties for fresh food preservation. Modified with permission from ref (284). Copyright 2023 Elsevier. (d) Schematic detailing production of anisotropic microcapsules via LbL assembly in a lithographic template sealed with biodegradable PLGA. (e) SEM micrographs of anisotropic capsules produced. Modified with permission from ref (274). Copyright 2022 Elsevier. (f) Probiotic-loaded flexible wood membrane used to form wooden scroll “capsule.” Modified with permission from ref (285). Copyright 2022 American Chemical Society.

In an attempt to tackle the accumulation of nonbiodegradable micro/nanoplastics in the environment, Liu and co-workers^275^ developed an ECHA-compliant microencapsulation technology for the sustained release of both aqueous soluble and insoluble actives, which relied on biodegradable silk fibroin. Encapsulation of model actives, such as herbicidal agents (e.g., saflufenacil), was attained by modulating silk protein protonation and its chain relaxation when self-assembly is induced through retrofit spray/ultrasonic freeze-drying techniques. This yielded engineered microcapsules with tunable morphology and topography, such as porous, smooth, and crumpled structures with a payload up to 50%. (Figure 12a-b) This payload was found to gradually release, with 20% being lost within the first 6 h; however, it required an additional 8.5 days for a total of 75% of the payload to be released. In addition to this, both release profile and biodegradability could be tuned by increasing the β-sheet wt% within the silk fibroin structure, demonstrating up to 65% degradation by mass over 10 days when placed in phosphate buffer solution (pH 7.4) at 37 °C containing protease XIV from *Streptomyces griseus*. These capsules may hold a potential for horticultural, food, and pharmaceutical applications. A relatively novel form of capsule formulation using amphiphilic polyelectrolyte to stabilize an oil in water emulsion has also been reported. The polyelectrolyte possesses increased amphiphilicity through the addition of a hydrophobic block grafted onto the polymer backbone.^276−278^ Consequently, the modified molecule contains a charged domain that may interact with the water and a hydrophobic block that interacts with the oil. Of particular interest is that polysaccharides may be modified to enable their use in this encapsulation technique. Glycidyl trimethylammonium chloride modified chitosan and hyaluronic acid have both been modified though grafting of a dodecyl alkyl chain and used for this purpose.^276−278^ Corn oil and oleic acid nano emulsions were used as a template to form capsules using modified hyaluronic acid and chitosan respectively.^278^ While traditional release studies were not performed, uptake of capsules to cancer cell lines demonstrated their ability to be effectively delivered to a target site. In addition to this, capsules demonstrated little degradation in storage at room temperature but were able to degrade in the presence of hyaluronidases explaining their ability to release cargo to a target cancer cell. However, the modification required to successfully implement this technique may also affect the biodegradability of the constituent polysaccharides. Thus, their use in more traditionally consumer-based products may not be viable until this potential issue is investigated.

These potential microcapsules are not restricted to the food and pharmaceutical sector and have been reported within the agricultural sector. Fu et al. were able to form microcapsules for the pesticide avermectin using 3,3′,4,4′-benzophenonetetracarboxylic dianhydride (BTDA) modified chitosan oligomer cross-linked by methane-4,4′-diisocyanate via interfacial polymerization to form a polyurea capsule.^279^ The BDTA was used to improve UV resistance of the microcapsule as avermectin has demonstrated sunlight sensitivity, which results in diminished pesticidal activity. These capsules exhibited sustained release rates over the course of approximately 1 week. Initial encapsulation in a polyurea shell demonstrated a 100% increase in the half-life of the pesticide. These capsules also demonstrated significant degradation when subjected to sunlight. These findings could be considered in combination with those reported by Wang et al.^280^ who encapsulated avermectin in PLA capsules of varying average diameters (344–827 nm). The capsules demonstrated significantly improved sustained release of avermectin over a period of 240 h when compared to bare avermectin and substantially decreased median lethal concentration (LC_50_) and photodegradation when compared to a currently available commercial product. Furthermore, decreasing the particle size increased the release rate due to increased interaction area/mass of the capsules with the medium in agreement with results by Li et al., who reported similar size correlations and improved UV resistance with increasing shell thickness.^281^ Combining these studies would lead to a potential capsule with controlled permeability and resistance to internalized active photodegradation able to be controlled via particle size and UV-resistant polymer loading while being able to tune the capsule photodegradation. However, the addition of the cross-linking agent may decrease the biodegradability of the polyurea capsule (which demonstrated significant photodegradation in simulated sunlight in water) must be considered in any potential final deployment.

Potential biodegradable polymer capsules with controllable release are not restricted to organic phase active ingredients. Aqueous core microcapsules were prepared by Abuhamdan et al.^282^ based on an internal phase separation method.^35,283^ PLGA and PLA polymer at varying quantities and ratios were dissolved in an oil phase followed by dropwise addition of water and emulsification in mineral oil using lecithin. Increasing the amount of water added during capsule preparation significantly decreased the encapsulation efficiency of a model active (fluorescein) for both PLA and PLGA capsules, in some cases as low as 10%. In addition, hydrophobic (due to ester termination and high lactide content) PLA capsules were able to demonstrate sustained release over 49 days with zeroth order release kinetics. However, PLGA capsules completely released their payload within 7 days. The release rates could be tuned by varying the ratio of PLA and PLGA – the latter being a biodegradable polymer. Increasing the PLGA content led to an increase in the release rate of the drug. While these capsules are not completely biodegradable the PLGA content could potentially be tuned to accomplish a required release rate, while maintaining a sufficient level of biodegradability to be compliant with incoming regulations.

Kudryavtseva et al.^274,286^ reported the fabrication of microprinted microcapsules made of PLA via an advanced soft lithographic technique. These capsules have since been further developed to incorporate a pH, thermo and ultrasonic release mechanism, to use PLGA as a biodegradable polymer capsule “lid”,^274^ and polyallylamine hydrochloride (PAH) and polysodium 4-styrenesulfonate (PSS) in LbL assembly instead of PLA (Figure 12d-e). The increase of surface area to volume ratio when compared to traditional spherical capsules may lead to a decrease in loss of active to environmental factors and increased loading. This is a result of an increased capsule-substrate contact area and consequent adhesion, resulting in less loss from capsules simply rolling off the target substrate. Furthermore, while currently only the “lid” of the capsule is formulated from biodegradable polymer, the work shows intent by these authors to both minimize waste and increase biodegradability in their capsule formulations–a promising start. The capsules were either pyramidally or rectangularly shaped and ranged in size from 1 to 11 μm, depending on the microprinting mold selected. Interestingly, the microcapsules were effective at retaining aqueous soluble molecules over a window up to a few days, making them potentially useful for intracellular drug delivery and other biocompatible applications. Overall, the shape and size of the microcapsules can be customized, and they can be designed to release their contents in response to specific stimuli.

Wang and co-workers developed polyurethane (PU) microcapsules that contain a blend of three essential oils (EOs). i.e. lavender essential oil, tea tree essential oil, and perilla leaf oil, to obtain a more harmonious aroma and increase the antibacterial activity of such microcapsules.^284^ The EOs@PU microcapsules were prepared via interfacial polymerization and had an average size of approximately 3 μm, which enabled high loading capacity of 59%. These microcapsules were incorporated into potato starch to produce food packaging films for sustained food preservation (Figure 12c). The films displayed low cell toxicity as well as excellent UV-blocking (>90%) properties. The sustained antibacterial efficacy of the starch-based packaging films due to the long-term release of the EOs@PU microcapsules extended the shelf life of fresh blueberries and raspberries at 25 °C (>7 days, when untreated berries were observed to visibly decay after 3 days). In addition, the biodegradation rate of these films after being cultured with natural soil was found to be 95% after 8 days. The excellent biodegradability of the starch-based packing films shows the potential of such films as an environmentally friendly, cost-effective and safe alternative to other films for sustained food preservation. Microencapsulation is also useful for protecting isocyanates from air moisture, increasing their storage stability as well as eliminating the hazards of direct handling.^287^ In the footwear industry, PU adhesives are usually coupled with isocyanate cross-linkers to accelerate the curing process, increase temperature resistance and enhance the endurance of the adhesive joint. Aguiar and co-workers reported a simple, effective and efficient approach for the encapsulation of isophorone diisocyanate (IPDI) within a biodegradable polymeric shell through a combination of an emulsion system with the solvent evaporation method. The microcapsules were spherical and composed of biodegradable polymer, i.e. poly(ε-caprolactone) (PCL), or blends of PCL and PLA as shell materials.^46,288^ High production yield (70–74%) and isocyanate loading of up to 73 wt % of the microcapsules were achieved using this approach. The resulting microcapsules had high retention and were capable of protecting the isocyanate core, particularly upon storage in low-moisture environments. Such microcapsules are a good candidate as cross-linking agents for adhesive formulations that are used in the footwear industry, and their application is not restricted by different legislations. Inorganic shell microcapsules, especially calcium carbonate, also present an effective route to long-term active retention. Work reported by both Zhao et al. and Keen et al.^289,290^ demonstrated the ability to store active ingredients for extended periods of time (between 1 and 6 months) with almost no loss, until acted upon by external stimuli. Zhao and co-workers produced microcapsules using water/oil/water double emulsions formed at 70 °C in a multilayered energetically efficient microfluidic device. The internal aqueous phase consisted of sodium carbonate which was pumped into a molten oil phase with a melting point of approximately 35 °C, this was then flowed through an outer aqueous poly(vinyl alcohol) solution and quickly cooled in an iced calcium chloride solution. This resulted in the oil layer being frozen and forming a solid shell. Due to the presence of the sodium and calcium salts in the internal and external phase, calcium carbonate precipitated when the phases met, effectively blocking any potential routes of leakage. However, when release was required, heating the microcapsule slurry and melting the oil allowed for complete release.^289^ Keen and colleagues utilized a traditional emulsification approach, forming a water in oil colloidosome using polymer latex particles (poly(methyl methacrylate)-co-butyl acrylate). In this work, sodium carbonate and amylase were encapsulated before being transferred into a continuous phase of calcium chloride, resulting in calcium carbonate precipitation. These microcapsules were able to preserve the enzyme and prevent leakage for nearly 6 months before being able to release the enzyme when subjected to shear comparable to that of a machine-washing cycle demonstrating their long-term stability and commercial potential.^290^ Furthermore, the capsules were largely comprised of materials that will readily degrade or could easily be replaced by materials that readily degrade. The oil phase reported by Zhao and co-workers was a mixture of fatty acids which could readily be replaced with edible or recyclable oils and the polymer latex presented by Keen and co-workers could be replaced with a biodegradable polymer such as PLGA.^291,292^

While not a traditional capsule, Luan et al. have developed an unusual and novel approach to active delivery.^285^ Their method relies on a pH modulated “unfolding” of a wood-based coil or scroll which has been loaded with an active. Basswood and balsawood were chemically treated with NaOH, Na_2_SO_3_ and boiling water to remove unwanted materials and render the wood flexible. This wood was then immersed in a solution containing *L. plantarum* – a rod-shaped probiotic for 16 h (Figure 12f). Following this the loaded wood was placed in a sodium alginate solution which was cross-linked using CaCl_2_. The alginate effectively acted as a glue to hold the wood scroll in shape until release. Release studies were performed with varying active ingredients such as rapeseed oil (nonaqueous) and tea polyphenols (aqueous). The probiotics were retained in an acidic environment simulating the stomach, but when in physiological pH simulating the intestinal tract, nearly 92% of the payload was released over 8 h in the case of the balsawood and 50% in the basswood demonstrating zero-order kinetics. This unique approach not only exploits the solubility and swelling of the alginate at higher pH to initiate release, but the unfolding kinetics reduces the risk of unwanted burst release as the active is slowly exposed to the neutral or alkaline environment. This highlights a dual approach seldom seen in the literature of combining structural changes (as opposed to ruptures or breakages) with chemically induced release mechanisms. Furthermore, all materials in this delivery device are naturally derived and degradable.

## 6 Conclusions/Outlook

While there has been an obvious shift in the design philosophy of encapsulation and microcapsules in recent years, the authors believe that the perfect microcapsule design has yet to be realized. To date, microcapsules formulated with fully biodegradable shells have not been demonstrated. This is even though many microcapsule shells are built from constituent polymers or inorganic materials that are biodegradable–the capsules themselves are rarely tested. In addition to this, microcapsule preparation via energetically effective processes is still an evolving area. Tunable release properties are certainly demonstrated in many microcapsule design examples, which are capable of retaining the encapsulated actives for various periods of time and able to precisely deliver them to target end-use sites. However, as far as the authors are aware, no single design has combined all these requirements into an ideal microcapsule.

In this article, we have reviewed the fundamental principles underpinning microcapsule formation including the impact and importance of material choice and release mechanisms. While polymers present simple routes for encapsulation and tuning via manipulation of polymer Mw, functional group and cross-linking density, the porous nature of polymer networks often results in permeable capsules. Although this permeability is acceptable to a certain extent in industry, indefinite long-term storage is likely not attainable. Conversely, inorganic or crystalline shells are more difficult to prepare but can offer improved mechanical and barrier properties due to their crystalline lattice, effectively trapping the encapsulated material within a robust shell. In addition, we have also provided some exemplar microcapsules to demonstrate how effective active ingredient retention can be attained with optimal shell formulation, such as composite and inorganic–organic hybrid microcapsules.

We have explored the most common release mechanisms (mechanical force/shear, ultrasound, pH and temperature response etc) and evaluated their effectiveness for their proposed purpose. Many capsules–especially those designed for drug delivery, are able to withstand the acidic digestive system, while releasing their payload in the intestinal tract where the pH is higher. In view of the advent of imminent legislation from the ECHA, forcing industry to adapt their designs to the new regulations but also opening new opportunities for considering additional shell materials and combination of materials, we have highlighted the relevant legal regulations, discussion papers and proposals put forward by the EU and how they likely affect current formulations. Furthermore, we have outlined how capsule biodegradability is assessed and discussed whether certain shell-forming materials can be in compliance with these impending new regulations, hence presenting potential alternative formulations.

We have also compared common emulsification techniques and highlighted their energy usage, a factor often overlooked within the literature–a key consideration as many microcapsules are obtained from emulsion templates.

Finally, we have highlighted some promising capsules that demonstrate a combination of excellent release/retention characteristics, low energy consumption, and biodegradability. Furthermore, we propose some suggestions for potential improvements within the existing microencapsulation systems.

On reflection of the current literature and state of the art, as well as impending international regulations, it is clear that the focus of microencapsulation in and beyond the consumer industry is changing. Efficiency, with respect to cost of manufacturing in the form of energy use and active ingredient cost is becoming a priority in both industrial and research sectors. Many research groups are currently striving to develop alternatives to traditional microencapsulation formulations, with emphasis on biodegradability, resource efficiency, and the circular economy. We believe that this can be achieved through a three-pronged approach:

First, encapsulation research should focus on biosourced or biodegradable materials. This may include forming microcapsules, with a combination of inorganic and organic materials (hybrid microcapsules), exploiting active ingredient physicochemical properties such as their solubility and adsorption properties and capitalising on the mechanical properties of both polymeric (flexibility, ease of encapsulation) and inorganic shells (improved mechanical/barrier properties and crystallinity).

A second point of focus should be to maximize active ingredient efficiency by optimizing microcapsule retention at the site of action. Two likely routes for achieving this are increasing chemical affinity of the microcapsule surface for the target site and tuning the morphology and mechanical properties of microcapsules by adjusting their shape, consequently offering higher microcapsule surface area for improved surface attachment. For capsules requiring adhesion to substrates, this may also result in a reduced overall active loss due to increased adhesion, and a reduction in waste. Similarly, premature release and loss of active ingredient due to mechanical rupture may be minimized by improving the mechanical properties of the microcapsule shells.

Finally, novel low-energy encapsulation methods should be explored. As outlined above, current emulsification methods are energy intensive or cannot be effectively scaled when preparing emulsion templates. To improve both commercial and environmental efficacy of capsule technology, additional methodologies should be investigated.

Despite encapsulation being a thriving industry for approximately half a century, there has been a recent need for improvement and optimization of microcapsule formulations to meet ongoing regulatory and industrial standards. It is our hope that this review has highlighted the key considerations and potential avenues for further progress within the microcapsule sectors both in the academic and industrial arenas.

Drawing on current knowledge, we trust that future microcapsule designs and microencapsulation processes will emphasize the integration of sustainable and advanced materials, preferably derived from natural, plant-based, and petroleum-free sources. However, researchers must be cognisant that capsule performance must be maintained in order for their implementation into consumer products. These innovations are poised to improve biodegradability, and ensure compliance with evolving regulatory standards, paving the way for safer and more efficient applications for the generations to come.
